# Development of a robust parallel and multi-composite machine learning model for improved diagnosis of Alzheimer's disease: correlation with dementia-associated drug usage and AT(N) protein biomarkers

**DOI:** 10.3389/fnins.2024.1391465

**Published:** 2024-09-06

**Authors:** Afreen Khan, Swaleha Zubair, Mohammed Shuaib, Abdullah Sheneamer, Shadab Alam, Basem Assiri

**Affiliations:** ^1^Department of Computer Application, Faculty of Engineering & IT, Integral University, Lucknow, India; ^2^Department of Computer Science, Faculty of Science, Aligarh Muslim University, Aligarh, India; ^3^Department of Computer Science, College of Engineering and Computer Science, Jazan University, Jazan, Saudi Arabia

**Keywords:** Alzheimer's disease, biomarker, early diagnosis, drug, hybrid clinical model, machine learning, multinomial classification, protein

## Abstract

**Introduction:**

Machine learning (ML) algorithms and statistical modeling offer a potential solution to offset the challenge of diagnosing early Alzheimer's disease (AD) by leveraging multiple data sources and combining information on neuropsychological, genetic, and biomarker indicators. Among others, statistical models are a promising tool to enhance the clinical detection of early AD. In the present study, early AD was diagnosed by taking into account characteristics related to whether or not a patient was taking specific drugs and a significant protein as a predictor of Amyloid-Beta (Aβ), tau, and ptau [AT(N)] levels among participants.

**Methods:**

In this study, the optimization of predictive models for the diagnosis of AD pathologies was carried out using a set of baseline features. The model performance was improved by incorporating additional variables associated with patient drugs and protein biomarkers into the model. The diagnostic group consisted of five categories (cognitively normal, significant subjective memory concern, early mildly cognitively impaired, late mildly cognitively impaired, and AD), resulting in a multinomial classification challenge. In particular, we examined the relationship between AD diagnosis and the use of various drugs (calcium and vitamin D supplements, blood-thinning drugs, cholesterol-lowering drugs, and cognitive drugs). We propose a hybrid-clinical model that runs multiple ML models in parallel and then takes the majority's votes, enhancing the accuracy. We also assessed the significance of three cerebrospinal fluid biomarkers, Aβ, tau, and ptau in the diagnosis of AD. We proposed that a hybrid-clinical model be used to simulate the MRI-based data, with five diagnostic groups of individuals, with further refinement that includes preclinical characteristics of the disorder. The proposed design builds a Meta-Model for four different sets of criteria. The set criteria are as follows: to diagnose from baseline features, baseline and drug features, baseline and protein features, and baseline, drug and protein features.

**Results:**

We were able to attain a maximum accuracy of 97.60% for baseline and protein data. We observed that the constructed model functioned effectively when all five drugs were included and when any single drug was used to diagnose the response variable. Interestingly, the constructed Meta-Model worked well when all three protein biomarkers were included, as well as when a single protein biomarker was utilized to diagnose the response variable.

**Discussion:**

It is noteworthy that we aimed to construct a pipeline design that incorporates comprehensive methodologies to detect Alzheimer's over wide-ranging input values and variables in the current study. Thus, the model that we developed could be used by clinicians and medical experts to advance Alzheimer's diagnosis and as a starting point for future research into AD and other neurodegenerative syndromes.

## 1 Introduction

Neurodegenerative diseases pose a significant challenge in contemporary medicine, presenting a substantial burden on healthcare systems worldwide and affecting the quality of life for millions globally (Whiteford et al., 2015). These disorders, exemplified by the gradual degeneration of the nervous system's structure and function, manifest in a myriad of ways, affecting cognition, motor skills, and overall neurological wellbeing. Neurological disorders affect roughly 15% of the global population at present (Feigin et al., 2020). Over the last three decades, the actual number of affected individuals has substantially increased.

After conducting a comprehensive analysis of various neurological conditions such as Alzheimer's Disease (AD), Huntington's Disease, Parkinson's Disease, and amyotrophic lateral sclerosis, dementia becomes evident as a significant outcome of neurological deterioration, with AD being the most prominent (Ritchie and Ritchie, 2012; Ciurea et al., 2023; Khan et al., 2023). Neurodegenerative disorders are multifaceted, and thus it is complex to diagnose since genetic, environmental, and age-related factors cause them.

Cognitive decline, a characteristic of AD and related dementias, encompasses a spectrum of cognitive impairments ranging from subtle changes in memory and thinking abilities to severe cognitive dysfunction affecting daily functioning (Whiteford et al., 2015; Feigin et al., 2020). Different etiologies contribute to cognitive decline, including neurodegenerative processes such as AD, vascular pathology, Lewy body disease, and other less common causes. Syndromic diagnosis, which includes subjective cognitive decline, mild cognitive impairment, and dementia stages of AD, plays a crucial role in characterizing the progression of cognitive decline (Ritchie and Ritchie, 2012; Ciurea et al., 2023).

Dementia currently has a staggering societal cost, accounting for 1.01% of global GDP (Mattap et al., 2022). This issue is expected to worsen in the coming years, with an estimated 85% increase in global societal costs by 2030, assuming no changes in potential underlying causes (e.g., macroeconomic aspects, dementia incidence and prevalence, treatment availability, and efficacy). As stated in the World Alzheimer Report 2023, the World Health Organization (WHO) warns of a rising global prevalence of dementia, which is anticipated to rise from 55 million in 2019 to 139 million by the year 2050. As societies continue to age, the related expenses of dementia are estimated to double, from $1.3 trillion in 2019 to $2.8 trillion by 2030 (Better, 2023).

AD poses a significant challenge to the global health landscape and is characterized by its relentless progression as a neurodegenerative disorder. The global rise in AD cases is directly linked to the aging population, with a rising number of people surviving above the age of 65 (the key age group prone to AD) (Jaul and Barron, 2017). The growing prevalence of AD in an aging population underscores the pressing need for a comprehensive understanding of the condition and the development of innovative diagnostic techniques (Saleem et al., 2022; Alqahtani et al., 2023). The defining characteristic of AD is the gradual deterioration of cognitive abilities, which ultimately compromises the quality of life for those affected. This disorder not only has a detrimental effect on memory but also disrupts various cognitive, behavioral, and daily functioning (Khan et al., 2023). The implications of this condition extend beyond those affected, impacting their families and caregivers and imposing a burden on healthcare systems globally.

The traditional diagnostic methods, however valuable, often fail to deliver timely and precise diagnoses. AD detection needs a more refined and advanced technique due to its complicated nature. Such an approach should not only involve identifying symptoms but also discerning underlying pathological changes in the brain. The current diagnostic framework for AD encompasses a comprehensive approach that combines clinical assessments, neuropsychological tests, neuroimaging methods, and biomarker analysis (Martí-Juan et al., 2020; Khan and Zubair, 2022a,b). Although these methodologies have provided valuable insights, there are still persistent problems, such as the need for early detection and the development of more accurate and reliable diagnostic tools. Thus, it is within this context that the role of protein biomarkers becomes crucial. Protein biomarkers, including Amyloid-Beta (Aβ), tau, and ptau [AT(N)] have emerged as a potential indicator in identifying the complex molecular and cellular alterations linked with AD (Martí-Juan et al., 2020; Khan et al., 2023). These biomarkers aid in the early detection of AD pathology, facilitating diagnosis at the MCI stage when interventions may be most effective. The incorporation of these indicators into diagnostic frameworks is consistent with the continued research of new strategies to address the challenges encountered by AD. However, diagnosis at preclinical stages, characterized by the absence of clinical symptoms, is not recommended in clinical practice and is primarily reserved for research purposes.

Machine learning (ML), a subfield of artificial intelligence, has emerged as a transformative technology in the healthcare industry, with the potential to change how neurodegenerative diseases are diagnosed and managed (Alowais et al., 2023). ML algorithms, equipped with the capacity to analyze vast datasets, recognize patterns, and derive meaningful insights, offer a paradigm shift in identifying subtle changes in neurological parameters that precede overt symptoms (Bhatia et al., 2022; Javaid et al., 2022). By assimilating information from multiple sources, for instance, neuroimaging, clinical data, and genetic profiling, ML algorithms contribute to the development of predictive models that aid in early detection and personalized treatment strategies (Jiang et al., 2017; Ahmed et al., 2020; Hossain and Assiri, 2020; Khan and Zubair, 2022a,b; Arafah et al., 2023; Assiri and Hossain, 2023).

In the present study, the optimization of predictive models for the diagnosis of AD pathologies was carried out using a set of baseline features, and the model performance was improved by incorporating additional variables associated with patient drugs and protein biomarkers into the model. Early AD was diagnosed by considering two key criteria: firstly, whether a patient was taking specific medications, and secondly, the presence of a significant protein serving as a predictor of Aβ, tau, and ptau levels among participants. In particular, we examined the relationship between AD diagnosis and the use of various medications (calcium and vitamin D supplements, blood-thinning medications, cholesterol-lowering drugs, and cognitive drugs). We also assessed the significance of three cerebrospinal fluid (CSF) biomarkers, tau, ptau, and Aβ in the diagnosis of AD. The relative importance of these biomarkers in diagnosing AD is still a topic of discussion in the academic community (Brookmeyer et al., 2007; Gauthier et al., 2021).

The adoption of a hybrid-clinical model, incorporating the simultaneous operation of multiple ML models in parallel, emerges as a viable strategy for enhancing predictive accuracy. Given the heterogeneous nature of the dataset under consideration, employing multiple ML models in parallel allows for a comprehensive classification approach. Subsequent to the individual classification outputs generated by each classifier, a majority voting mechanism is employed to aggregate predictions. This collective decision-making process, leveraging the consensus among classifiers, serves to enhance overall predictive accuracy. Notably, the incorporation of parallelization principles within our model framework not only contributes to improved performance but also facilitates efficiency gains by optimizing computational resources.

The proposed model is used to simulate the MRI-based data, with five diagnostic groups of individuals (cognitively normal, significant subjective memory concern, early mildly cognitively impaired, late mildly cognitively impaired, and AD), with a further refinement which includes preclinical characteristics of the disorder. It is noteworthy that we aimed to construct a pipeline design employing ML that incorporates comprehensive methodologies to detect Alzheimer's over a wide- ranging input values and variables in the current study. The proposed design builds a meta-model based on four distinct sets of criteria, which include diagnosing from baseline features, baseline and medication features, baseline and protein features, and baseline, medication, and protein features. The meta-model incorporated a 4-step data preprocessing strategy, followed by feature wrapping using the step-forward technique. Furthermore, twelve efficient ML algorithms served as base classifiers. During the construction of the hybrid model, both stacking and voting techniques were employed. Preceding this, cross-validation with 5 and 10 folds was implemented alongside hyperparameter optimization. Subsequently, performance evaluation and comparison were conducted based on various metrics.

Thus, this research seeks to contribute to the broader effort of improving diagnostic approaches for Alzheimer's. This study aims to develop a robust multi-composite machine learning model that improves diagnostic accuracy by studying the intricate relationship between protein biomarkers, drugs, and AD. The model that we have developed offers a tool for healthcare practitioners to advance Alzheimer's diagnosis while also laying the groundwork for further investigation into AD and other neurodegenerative conditions.

## 2 Methods

### 2.1 Proposed design

**Figure 1:**
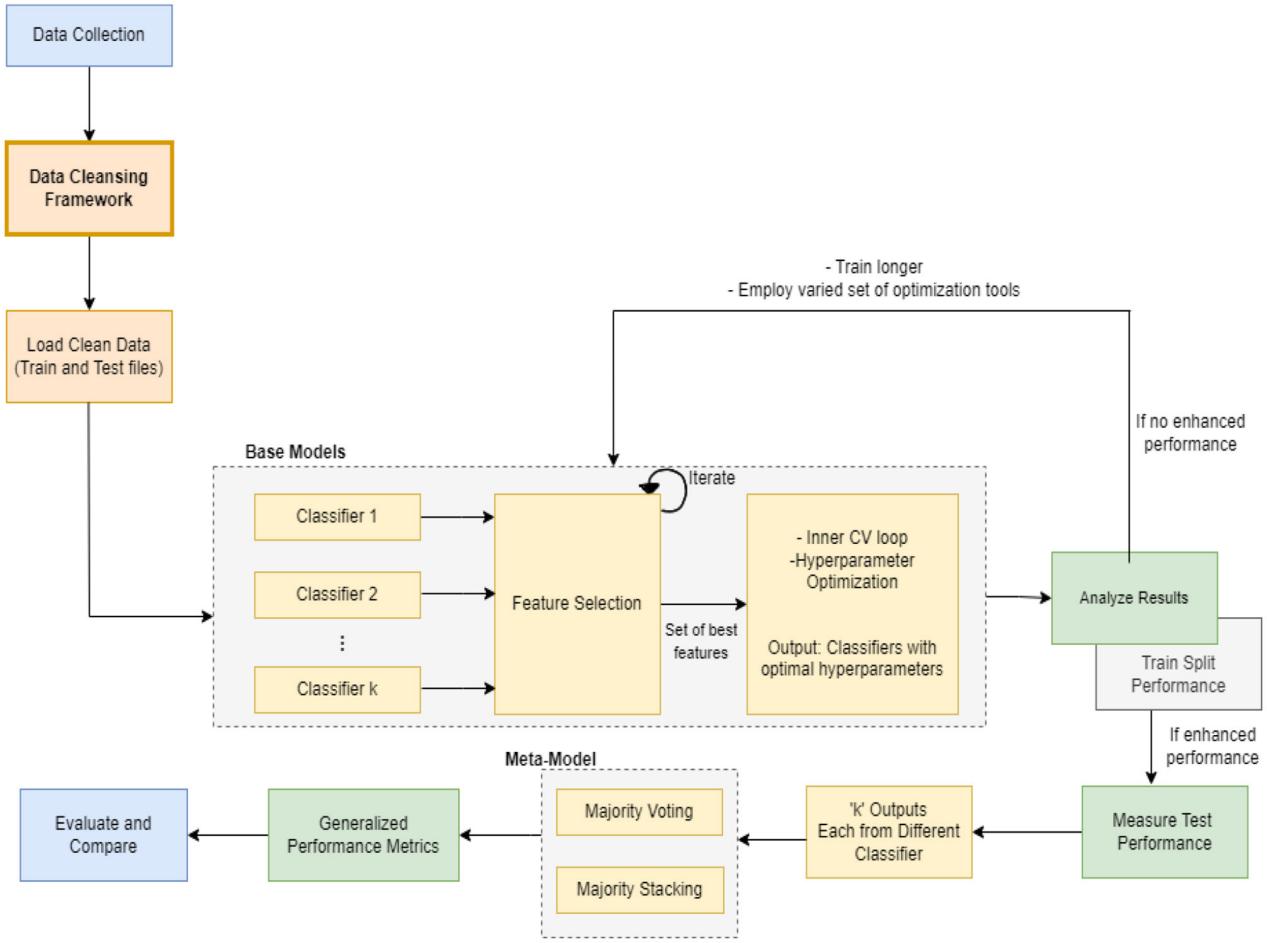
Hybrid-clinical model architecture.

**Figure 2:**
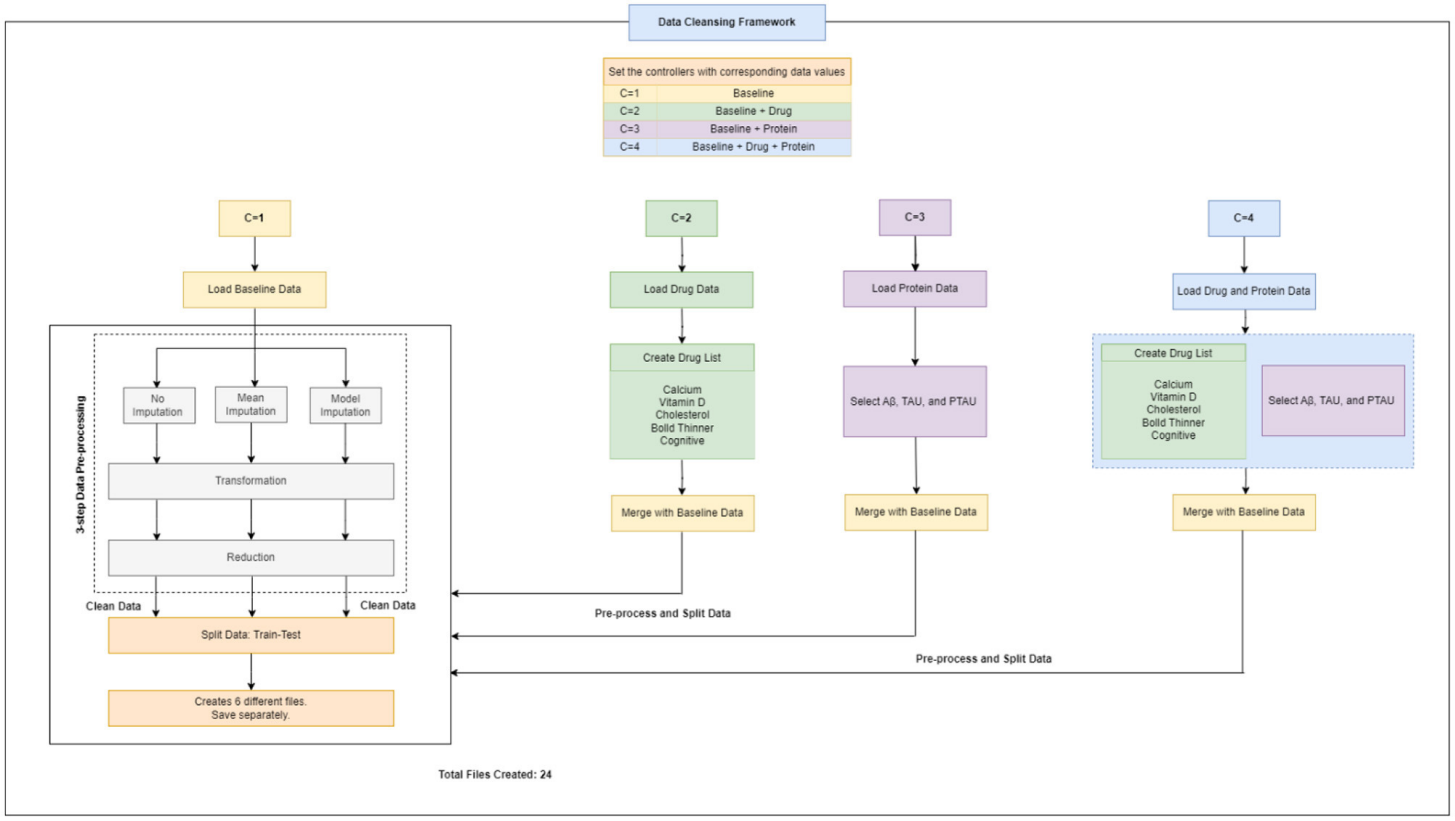
Workflow design for data cleansing framework.

In this section, we present the proposed meta-model, followed by an outline of the key steps involved in our method. The purpose of creating a pipeline environment is to streamline the entire process and ensure that the procedure is successful, i.e., to facilitate internal verification and produce outcomes that are reproducible externally. A schematic flow of our end-to-end approach is illustrated in Figures 1, 2. In the subsequent sections, a detailed description of the proposed approach and the various steps undertaken in this study are presented.

Every machine learning model possesses unique characteristics that enable it to achieve success with specific types of data. As this task involves various kinds and groups of data, we suggest utilizing multiple machine learning models in parallel to classify the data. Once we obtain the output of each classifier, which is a specific class, we aggregate the majority vote of the classifiers to improve overall accuracy. Our model employs the principle of parallelism to increase system accuracy and expedite processing time.

### 2.2 Study design, participants and dataset collection

Alzheimer's Disease Neuroimaging Initiative (ADNI) is a comprehensive repository that was established in 2004 and is headed by Michael W. Weiner as the principal investigator.^1^ This repository contains data on clinical, biochemical, genetic, and imaging biomarkers for detecting AD and MCI at an early stage, monitoring their progression, and tracking their development over time through a series of longitudinal, multicenter studies.

The baseline statistical model that we designed was based on the ADNIMERGE dataset from ADNI, which contained selected factors relevant to individuals' clinical, genetic, neuropsychological, and imaging results. The ADNIMERGE dataset consists of four distinct studies, namely ADNI-1, ADNI-2, ADNI-3, and ADNI-GO, which were collected at varying stages of the research project and represent different time periods. Each dataset includes new patients who were enrolled during the study period, as well as previous patients who were continuously monitored. The ADNIMERGE dataset includes 2,175 individuals, ranging in age from 54 to 92 years, and contains 14,036 input values for 113 features. These values were collected over a period of 8 years (2004–2021), with the initial measurement taken when the patient first arrived, followed by a 6-month follow-up visit every year for 8 years. We selected only patients who participated in the ADNI-1 phase of the initiative, which comprised 818 individuals and a total of 5,013 input values across 113 variables. These participants were characterized by demographic information, neuropsychological, genetic, MRI, Diffusion-tensor imaging (DTI), electroencephalography, and positron emission tomography (PET) biomarkers. This was done in order to maintain uniformity across studies and data handling, as well as to ensure that we could successfully select only a single observation for each subject.

This study included and integrated three varied datasets i.e., baseline data, drug data and protein biomarker data. Separate data files containing drug and protein biomarker data were stored on the ADNI repository. They were examined to extract the pertinent ones and were added to the baseline data.

#### 2.2.1 Baseline dataset

The baseline dataset comprised 818 participants from the ADNI-1 dataset. given our objective of using predictors that have been consistently evaluated and effectively integrated into clinical settings, while also being non-intrusive to patients, we have chosen to exclusively utilize variables from the ADNI-1 baseline dataset. these variables pertain to diagnostic subtypes, demographic details, and scores from clinical and neuropsychological tests. only diagnoses that were confirmed from screening up until the baseline visit were considered, whereas any patient data with more than 20% missing information were eliminated.

Based on the diagnosis at the follow-up visit, the patients who were diagnosed at baseline were classified into 5 distinct categories: Cognitively Normal, Early Mild Cognitive Impairment, Late Mild Cognitive Impairment, Significant Memory Complaints, and Alzheimer's Disease, abbreviated as CN, EMCI, LMCI, SMC, and AD. The diagnostic classes EMCI and LMCI were unified into a single diagnostic class MCI. An additional advantage of doing so was that it made it easier to extend our model in the future for analyses that might contain additional ADNI data. Because SMC patients met the criteria for being cognitively normal, the CN and SMC classes were combined into a single category. The diagnosis features, which served as a response variable, were thus divided into 3 categories: AD, MCI, and CN. Among the 818 patients involved in the study, 193 individuals were identified with AD, 396 with MCI, and 229 with CN. The demographic information of the study participants, categorized by their baseline diagnosis, is presented in Table 1. Age, years of education, gender, marital status, ADAS11, ADAS13, ADASQ4, CDRSB, DIGITSCOR, FAQ, LDETOTAL, MMSE, mPACCdigit, mPACCtrailsB, TRABSCOR, RAVLT-I, RAVLT-L, RAVLT-F, and RAVLT-PF, are the final variables considered in the present study. The statistics and descriptions of these variables are provided in Table 2.

**Table 1:** Subject demographics.

**Attribute**	**Total subjects: 818 Male: 476 (58.20%) | Female: 342 (41.80%)**		
	**AD**	**MCI**	**CN**
Gender (M|F)	102 (52.84%) | 91 (47.15%)	255 (64.39%) | 141 (35.61%)	119 (51.96%) | 110 (48.03%)
**Age**			
Range (Mean | SD)	55.1-90.9 (75.28 | 7.45)	54.4-89.3 (74.43 | 7.40)	59.9-89.6 (75.84 | 5.02)
Education (Years) Range	4 - 20	4 - 20	6 - 20
**Marital Status**			
Married | Never Married | Divorced | Widowed | Unknowns	157 | 7 | 9 | 20 | 0	317 | 6 | 25 | 48 | 0	156 | 15 | 17 | 40 | 1
**Ethnicity**			
Hispanic/Latin | Non-Hispanic/Latin | Unknowns	4 | 187 | 2	13 | 380 | 3	2 | 226 | 1
**Race**			
White | Black | Asian | Indian/ Alaskan | More than 1 type	181 | 8 | 2 | 0 | 2	370 | 15 | 9 | 1 | 1	210 | 16 | 3 | 0 | 0

**Table 2:** Variable description and related statistics.

**Variables**		**AD Mean (SD)**	**MCI Mean (SD)**	**CN Mean (SD)**	**% Missing at baseline**
**ADAS** (Alzheimer's Disease Assessment Scale) A comprehensive assessment to identify cognitive and non-cognitive signs of Alzheimer's.	*ADAS11 (Alzheimer's Disease Assessment Scale - 11 items)* This evaluation consists of 11 questions. The score range for these questions is between 0 and 70. A score of 0 represents no impairment, whereas a score of 70 indicates considerable impairment.	18.60 (6.28)	11.4 (4.42)	6.20 (6.20)	0.12
	*ADAS13 (Alzheimer's Disease Assessment Scale - 13 items)* This test consists of 13 questions, and the score ranges from 0 to 85. A score of 0 signifies no impairment, whereas a score of 85 signifies significant impairment.	28.87 (7.62)	18.62 (6.27)	9.50 (4.19)	0.97
	*ADAS4* This is task 4 in ADAS11. It is the cognitive subscale for word recognition.	8.56 (1.56)	6.18 (2.26)	2.85 (1.72)	0.0
**CDR-SB** (Clinical Dementia Rating–Sum of Boxes)	It assesses the progression of dementia, particularly in people with mild to moderate cognitive decline. A semi-structured interview is conducted with the patient and other interviewees, such as family members, to obtain the rating. The range of values spans 0 to 18.	4.29 (1.64)	1.60 (0.88)	0.03 (0.12)	0.0
**DIGITSCOR** (Digit Span Test Score)	This test is performed to determine the storage capacity of a number. Participants are given a number sequence and instructed to repeat it back to the assessor in either forward or reverse order.	26.93 (12.81)	36.85 (11.17)	45.75 (10.20)	0.61
**FAQ** (Functional Assessment Questionnaire)	It evaluates a patient's capacity to independently perform routine tasks. The scale ranges from zero to thirty. A score of 0 signifies normal, whereas a score of 30 shows that the individual is excessively dependent.	12.99 (6.84)	3.82 (4.46)	0.14 (0.60)	0.36
**LDETOTAL** (Delayed Total Recall)	It is a neuropsychological test that assesses an individual's capacity to recall information after a certain period of time.	1.27 (1.90)	3.81 (2.27)	12.97 (3.57)	0.0
**MMSE** (Mini-Mental State Examination)	It is a questionnaire-based evaluation designed to detect cognitive impairment. It has a range from 0 to 30. Normal scores range from 25 to 30; mild scores range from 21 to 24, moderate scores from 10 to 20, and severe scores from 0 to 10.	23.34 (2.06)	27.03 (1.78)	29.11 (0.98)	0.0
**mPACC** (Modified Preclinical Alzheimer Cognitive Composite) Cognitive abilities, timed executive function, and episodic memory are evaluated by these tests.	*mPACC-digit (Modified Preclinical Alzheimer Cognitive Composite with Digit)* This mPACC test utilizes digit substitution.	−13.98 (3.01)	−7.47 (3.29)	−0.12 (2.47)	0.0
	*mPACC-trailsB (Modified Preclinical Alzheimer Cognitive Composite with Trails B)* This mPACC test employs Trails B substitution.	−14.24 (3.09)	−7.60 (3.39)	−0.33 (2.44)	0.0
**RAVLT** (Rey Auditory Verbal Learning Test) RAVLT is a neuropsychological test that is commonly used to assess auditory-verbal abilities such as attention, memory, and learning ability. The RAVLT is a five-trial process (Trials 1-5) that consists of presenting a list of fifteen words. Following 30 minutes of interpolated testing, the participant is asked to recall the terms from the first set. This is known as delayed recall. These scores are then used to calculate various summary scores.	*RAVLT-L (Rey Auditory Verbal Learning Test – Learning)* It is derived by subtracting the Trial 1 and Trial 5 scores.	1.81 (1.79)	3.30 (2.35)	5.85 (2.28)	0.48
	*RAVLT-I (Rey Auditory Verbal Learning Test – Immediate)* It is calculated by adding the results from the first five trials (Trials 1–5).	23.16 (7.70)	30.76 (9.04)	43.33 (9.09)	0.48
	*RAVLT-F (Rey Auditory Verbal Learning Test – Forgetting)* It is derived by subtracting the Delayed Recall score from the Trial 5 score.	4.54 (1.91)	4.67 (2.26)	3.58 (2.73)	0.48
	*RAVLT-PF (Rey Auditory Verbal Learning Test - Percent Forgetting)* It is derived by dividing the RAVLT-F score by the Trial 5 score.	88.70 (21.92)	67.86 (31.41)	34.18 (27.64)	0.97
**TRABSCOR** (Trail Making Test Part B Time)	This diagnostic assessment evaluates cognitive functioning, specifically the capacity for reasoning, information retention, and thought.	197.95 (87.09)	130.74 (73.69)	89.21 (44.26)	1.71

#### 2.2.2 Drug dataset

We created five new variables from components inside the drug dataset that fall into one of our five analytic categories: blood thinners, calcium doses, cholesterol-lowering medicines, cognitive drugs, and vitamin d medicines. Table 3 contains a comprehensive list of individual drug and supplement names. it is critical to note that the use of any of these drugs did not preclude a patient from participating in the ADNI cohort (see footnote^1^).

**Table 3:** Drug list.

**Supplement type**	**Supplement name**
Blood thinners	Dabigatran, Naprapac, A.P.C., A.S.A., Actron, Advil, Aggrenox, Aleve, Alka-Seltzer, Anacin, Anaprox, Anexsia, Anodynos, Ansaid, Apixaban, Arthritis, Artrotec, Ascriptin, Aspergum, Aspirin, Axotal, Bac, Bayer, Bexophene, Bextra, Biloba, Brilinta, Buffaprin, Buffered, Bufferin, Buffinol, Cama, Cataflam, Celebrex, Cheracol, Clinoril, Clopidogrel, Combunox, Compound, Congespirin, Coumadin, Damason Darvon, Dasin, Daypro, Dhc Diagesic, Diclofenac, Dipyridamole, Disalcid, Dolabid, Dolprin, Doxaphene, Dristan, Easprin, Ecotrin, Eliquis, Emagrin, Empirin, Equagesic, Equazine, Etexilate, Etodolac, Excedrin, Feldene, Fenoprofen, Fiogesic, Fiorgen, Fiorinal, Forte, Gemnisyn, Ginko, Heparin Ibuprofen, Indocin, Indomethacin, Joseph, Ketoprofen, Ketorolac, Liquprin, Lodine, Lortab, Magnaprin, Marnal, Measurin, Meclofenamate, Mefenamic, Meloxicam, Meprobamate, Midol, Mobic Momentum, Motrin, Nabumetone, Naprapac, Naprelan, Naprosyn, Naproxen, Nasal Norgesic, Nuprin, Orudis, Oruvail, Oxaprozin, P, Pabalate, Percodan, Persantine, Persistin, Pf Piroxicam, Plavix, Pletal, Plus, Ponstel, Pradaxa Presalin, Prevacid Profen, Relafen, Rivaroxaban, Robaxisal, Roxiprin, Rufen, Saleto, Salocol, Salsalate, Soma Spray, Sprix, St., Sulindac, Supac, Synalgos, Talwin, Ticagrelor Ticlid, Ticlopidine Tolectin, Tolmetin, Toradol Trental, Trigesic, Trilisate, Ultraprin, Unipro, Vanguish, Vicoprofen, Vimovo, Voltaren, Warfarin, Xarelto, Zipsor, Zorpin
Calcium	Calcium (No specific drug name was there in the dataset; only the “calcium” term was used.)
Cholesterol-lowering	Altoprev, Atorvastatin, Crestor, Fluvastatin, Lescol, Lescol Xl, Lipitor, Livalo, Lovastatin, Mevacor, Pitavastatin, Pravachol, Pravastatin, Rosuvastatin, Simvastatin, Zocor
Cognitive	Aricept, Donepezil, Exilon, Galantamine, Memantine, Namenda, Namzaric Razadyne, Rivastigmine
Vitamin D	Vitamin D (No specific drug name was there in the dataset; only the “vitamin D” term was used.)

#### 2.2.3 Protein biomarker dataset

Using the ADNI dataset, we generated three new variables from components within the CSF biomarker data to determine the amounts of Aβ, tau, and ptau.

Therefore, altogether three unique datasets (baseline data, drug data, and protein data) were gathered, extracted and handled, and then were passed to the next step i.e., data cleansing framework for data pre-processing (Figure 2).

### 2.3 Data cleansing framework

A flowchart depicting the process of complete data cleansing is portrayed in Figure 2. Initially, the controllers were configured with specific data values, including C = 1 for baseline data, C = 2 for baseline and drug data, C = 3 for baseline and protein data, and C = 4 for baseline, drug, and protein data, respectively. Table 4 shows a complete description of the procedure for implementing the four sets of controllers. After the execution of the data cleansing process, 24 individual clean files were generated.

**Table 4:** Execution steps for data cleansing framework.

**When C = 1**, • load only the baseline data. • pre-process the data using a 3-step ITR strategy (described below) • split into train test data; this will create six different clean files (train and test files for without imputation technique, train and test files for mean imputation, and train and test files for model imputation technique)	**When C = 2**, • load only the drug data. • merge with the original baseline data • pre-process the data using a 3-step ITR strategy. • split into train test data; this will create six different clean files (train and test files for without imputation technique, train and test files for mean imputation, and train and test files for model imputation technique)
**When C** **=** **3**, • load only the protein biomarker data. • merge with the original baseline data. • pre-process the data using a 3-step ITR strategy. • split into train test data; this will create six different clean files (train and test files for without imputation technique, train and test files for mean imputation, and train and test files for model imputation technique)	**When C** **=** **4**, • load drug and protein biomarker data. • merge with the original baseline data. • pre-process the data using a 3-step ITR strategy. • split into train test data; this will create six different clean files (train and test files for without imputation technique, train and test files for mean imputation, and train and test files for model imputation technique)

**3-step data pre-processing strategy:**

The dataset acquired was processed with a three-step ITR approach i.e., **Imputation (I), Transformation (T)**, and **Reduction (R)**.

#### 2.3.1 Imputation

In this study, the dataset encountered issues of noise, incompleteness, and inconsistency, which typically hinder the mining process. In general, inaccurate or dirty data often pose challenges for mining techniques, which can obstruct the extraction of valuable insights. The proportion of missing values for the extracted variables (at baseline) is depicted in Table 2. To overcome the challenge of missing data, there exists various techniques. Specifically in this study, three different approaches were employed to address this issue. The simplest method involved removing all instances of missing data, which was implemented as the first strategy, referred to as “without imputation.”

There are various methods for handling missing data, including weighting, case-based, and imputation-based techniques (Tartaglia et al., 2011). The latter technique was used in the present study. Imputation involves predicting missing data values and then filling them with suitable approximations, such as the mean, median or mode. Subsequently, standard complete-data techniques are then applied to the filled-in data to decrease the biases due to missing values and improve the efficiency of the model.^2^ The term “mean imputation” refers to replacing missing values with suitable approximations, such as the mean, and subsequently applying standard complete-data procedures to the filled-in data (Khan and Zubair, 2019). This was the second approach employed in the study. The “model imputation” method, on the other hand, involves replacing missing values with appropriate approximations, such as a linear regression model, and then using a standard complete-data process to the filled-in data (Khan and Zubair, 2019). This was the third approach used in the study.

#### 2.3.2 Transformation

In this study, data transformation involved two main techniques: normalization and smoothing. These techniques aim to improve the quality and interpretability of the data (Pires et al., 2020; Maharana et al., 2022). In this study, normalization was applied to the ADNI dataset to standardize the scale of numerical values, which varied in range for different variables. The values were adjusted and transformed in a way that they fall within a specified range, often between 0 and 1 (Pires et al., 2020; Maharana et al., 2022). This adjustment ensured that each variable had equal importance in the analysis and prevented any one variable from dominating due to its scale. Smoothing was then performed to remove any noise or irregularities in the ADNI data, making it easier to identify underlying patterns. This was particularly useful for our research, as the data contained random variations and anomalies that could have masked meaningful trends and patterns.

#### 2.3.3 Reduction

Data reduction methodologies play a significant role in the analysis of reduced datasets while maintaining the integrity of the original data (Khan and Zubair, 2022a). This approach is often used to enhance efficiency, streamline analysis, and effectively manage large datasets (Maharana et al., 2022). There are several techniques for implementing data reduction i.e., dimension reduction, sampling, aggregation, and binning. Each method is applied based on the type of dataset and variables present. In this study, we employed a dimension-reduction strategy. This method facilitated in identifying and eliminating variables and dimensions that were insignificant, poorly correlated, or redundant.

### 2.4 Load clean data

The subsequent step involved loading the clean data (as depicted in Figure 1). Initially, the clean data files generated when the controller was set to 1 were loaded and the entire pipeline was executed. Similarly, this process was repeated for the remaining three controllers, 2, 3, and 4, resulting in the creation of twelve different ML meta-models. Later, comparisons were performed to determine which model performed optimally across a range of applied methods.

### 2.5 Machine learning modeling

Machine learning techniques were utilized to develop a classifier capable of identifying potential instances of AD and MCI. A hybrid-clinical classification model was constructed, incorporating variables selected during the feature selection process. This model development process was repeated for each of the 4 controllers separately. The clean data was passed to the base ML classifiers. The model was trained on the training set. The feature selection process was conducted to determine the optimal set of features. The performance was assessed, and it was made to run iteratively until the best feature set was identified. Subsequently, we applied 5-fold repeated stratified cross-validation and hyperparameter optimization techniques to obtain an optimized set of algorithms. The optimized classifier, trained on the complete training set, was then applied to the independent test set following 10 iterations of 5-fold repeated stratified cross-validation on the training set.

**Figure 3:**
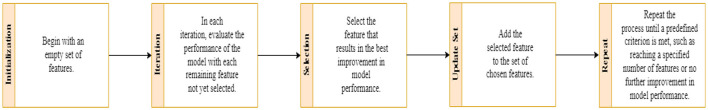
Five-step process: step forward feature selection.

**Figure 4:**
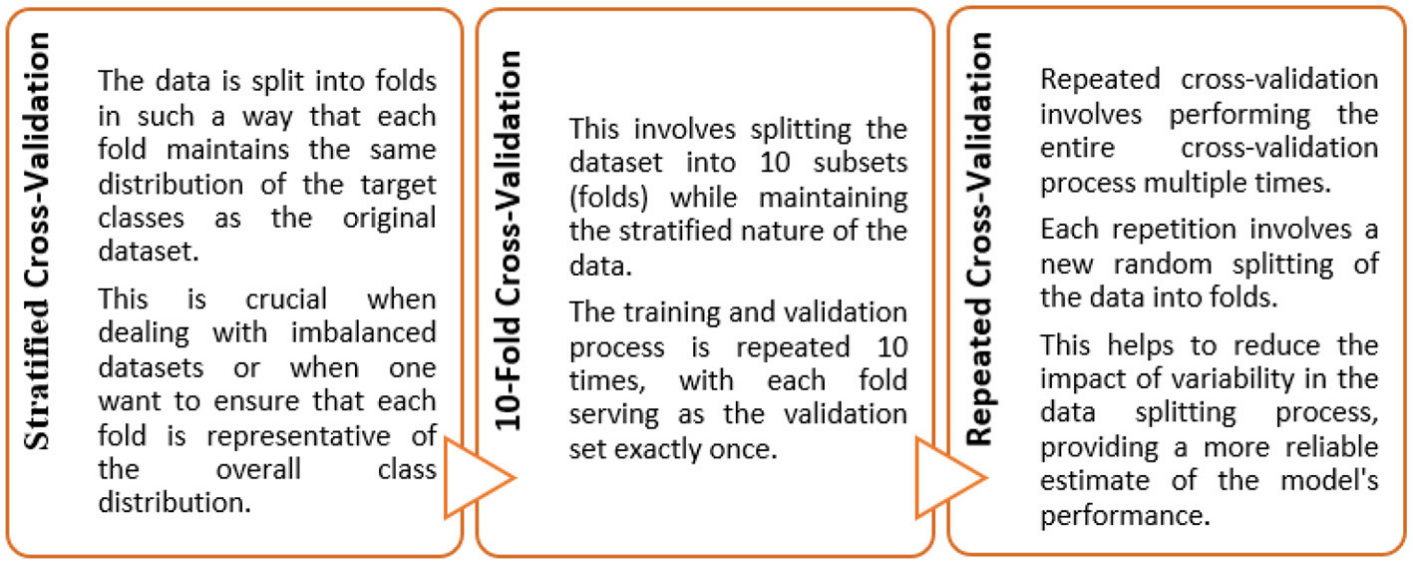
Ten-fold repeated stratified cross-validation.

The entire modeling process is explained in the following sections and can be seen in Figures 1, 3, 4 pictorially.

#### 2.5.1 Base ML models

We used a varied set of ML algorithms and techniques in our operations. A good implementation of the algorithms in question was known while selecting these tools for this study. In this study, twelve efficient ML models were used, namely, Multinomial Logistic Regression, K-Nearest Neighbors, Linear Discriminant Analysis, Quadratic Discriminant Analysis, Decision Tree, Random Forest, AdaBoost, Principal Component Analysis with Logistic Regression, Support Vector Machine - Radial Basis Function, Perceptron, MultiLayer Perceptron, and Elastic Nets. Earlier, fifteen supervised learning classifiers were executed to study the impact of the built model. However, we selected twelve classifiers out of these fifteen classifiers. Gaussian Process, LinearSVC and Stochastic Gradient Descent are the three ML algorithms that could not fit on the ADNI dataset efficiently and hence resulted in reduced performance. These 12 models were chosen for their ability to produce high performance during the model development step. These models acted as the base ML models for the creation of a hybrid meta-model.

The decision to use twelve ML classifiers out of fifteen can be attributed to the thorough consideration of algorithm performance and effectiveness in this study. The selection of these twelve classifiers was based on the following factors:

- Diverse Set of Algorithms: The initial set of fifteen classifiers likely encompassed a diverse range of ML algorithms, allowing for comprehensive coverage of different approaches. By including a variety of models, the study aimed to explore the strengths and weaknesses of various algorithms, ensuring a more robust understanding of the data and problem domain.
- Performance Evaluation: Initially, we explored fifteen supervised learning classifiers. The decision to narrow down to twelve models suggests that an extensive evaluation was conducted, and the performance of each algorithm was thoroughly assessed. Only the top-performing models were retained for further analysis.
- Elimination of Underperforming Models: The decision to exclude certain models was based on their underperformance and lack of contribution to achieving the study's objectives, emphasizing the importance of selecting the most effective algorithms.

The description of each employed classifier is presented below:

A. Multinomial Logistic Regression (MLR): In binary logistic regression, we estimate the probability of a single class, while in MLR, we extend this concept to estimate probabilities for multiple classes. This approach is particularly effective for dependent variables with three/more unordered categories. MLR applies a logistic function to each category to calculate the probability of belonging to that specific group, with k categories are represented by k-1 logistic functions (Khan et al., 2024). To interpret the results in terms of relative likelihoods, the probabilities are then normalized to ensure that they sum to 1 across all categories (Aguilera et al., 2006; Yang, 2019). MLR calculates a set of coefficients for each category, representing the correlation between predictor variables and log-odds of belonging to that group (Yang, 2019; Reddy et al., 2024). Each category has its intercept, which represents the log-odds of all predictor variables being zero. The MLR is usually trained using maximum likelihood estimation, where the model parameters are calculated to maximize the likelihood of observing the specific set of outcomes (Hedeker, 2003).
B. K-Nearest Neighbors (KNN): The KNN algorithm is a supervised ML technique that is primarily used for classification. The main concept of KNN is to generate predictions by examining the majority class amongst the “K” nearest data points. In the context of classification, when presented with a new data point, KNN examines the “K” data points in the training set that are in closest proximity to it (Li, 2024). The class with the highest prevalence among these nearby data points is then allocated to the new data point. Typically, Euclidean distance (a distance metric) is employed by KNN for measuring the similarity or proximity of data points within a multi-dimensional feature space (Zhang et al., 2022; Li, 2024). The parameter “K” indicates the total number of neighbors to take into account. The choice of an appropriate “K” is essential as it can significantly impact the quality of predictions. A small value of K can result in noisy predictions, whereas a larger value of K can help to identify underlying patterns more accurately (Li, 2024). The KNN classifier identifies a set of neighbors and then counts the number of instances of each class amongst these neighbors. The class with the most occurrences is then assigned to the new data point. This is an instance-based learning algorithm that memorizes the training instances rather than explicitly learning a model. KNN is a non-parametric method which means it makes no assumptions regarding the underlying distribution of the data (Wang et al., 2022).
C. Linear Discriminant Analysis (LDA): It is a supervised algorithm designed to discover the optimal linear combinations of features that efficiently separate different classes within a dataset (Tharwat et al., 2017). By maximizing the separation between these classes, LDA effectively results in the formation of different groups of data points (Seng and Ang, 2017). The algorithm intrinsically performs dimensionality reduction, which is an inherent feature of the algorithm. This involves projecting the data onto a lower-dimensional space while preserving the most informative features for classification (Seng and Ang, 2017; Graf et al., 2024). LDA makes the assumption that the covariance matrix is consistent across all classes and that the features within each class follow a normal distribution (Seng and Ang, 2017; Tharwat et al., 2017). To effectively distinguish the distribution of data points within and between classes, LDA computes mean vectors and scatter matrices. Mean vectors represent the centroids of data points in each class, whereas scatter matrices capture the spread or variability within each class. LDA then performs eigenvalue decomposition on the generalized eigenvalue problem that includes within-class and between-class scatter matrices (Tharwat et al., 2017). The directions of maximum discrimination are revealed by the eigenvectors that correspond to the largest eigenvalues. The data points are then projected onto these discriminant directions, resulting in a new space where the classes are clearly separated (Tharwat et al., 2017).
D. Quadratic Discriminant Analysis (QDA): It is a supervised classification algorithm that aims to determine the optimal boundaries for separating different classes in the feature space (Jiang et al., 2018). In contrast to LDA, QDA provides greater flexibility in capturing variability within each class by allowing distinct covariance matrices for each class (Witten et al., 2005; Tharwat et al., 2017). QDA, analogous to LDA, aims to maximize the degree of segregation among distinct classes. This is achieved through the identification of quadratic decision boundaries, which adequately represent the complex relationships among the features (Witten et al., 2005). Because LDA presumes that all classes utilize the same covariance matrix, QDA allows each class to possess a unique covariance matrix. Thus, QDA is more adaptable when handling classes that may exhibit diverse variability patterns (Siqueira et al., 2017). Similar to LDA, QDA calculates the mean vector for each class that serves as the centroid of the data points in that class. In addition, QDA determines the scatter matrices for each class, which reflect the dispersion or variability present within each class (Witten et al., 2005). Following that, it sets up quadratic decision boundaries that effectively separate classes using data from the class means and scatter matrices. The function of the decision boundaries is to classify the newly acquired data points. Compared to LDA, QDA is better at finding non-linear correlations and complex patterns within each class because it uses different covariance matrices (Siqueira et al., 2017).
E. Decision Tree (DT): A Decision Tree classifier is a tree-like model that follows a hierarchical structure, where input data points are classified based on a series of decisions made at each node (Khan and Zubair, 2022a,b). Leaf nodes represent the predicted class, whereas internal nodes reflect decisions based on particular features. The tree structure is composed of nodes that learn the most important features for classification. In DT, the features that provide the best separation of classes are positioned closer to the root of the tree (Blockeel et al., 2023; Costa and Pedreira, 2023). The root node is the top node in the hierarchy. It represents the complete dataset and is divided according to the feature that best separates classes. The process of selecting features and splitting the dataset recursively continues until a stopping criterion is met (Blockeel et al., 2023; Costa and Pedreira, 2023). When a new data point traverses the tree, it follows the decision path based on the conditions of the features until it gets to a leaf node. The predicted class for the input data point is then determined by the class associated with that leaf node.
F. Random Forest (RF): Random Forest is an ensemble learning ML model that aggregates predictions from multiple ML models to construct a more robust and precise model (Belle and Papantonis, 2021). It employs decision trees as its base model and uses a bagging technique, which trains several trees on various subsets of the training data (Campagner et al., 2023). For classification, the final prediction of the RF algorithm is determined through a voting mechanism. Each tree votes for a class and the class with the most votes is assigned to the input data point. RF produces multiple subsets of training data using random sampling with replacement (bootstrap sampling). Each subset trains a distinct DT. For each DT, a feature subset is randomly selected at each split. This approach guarantees that the generated trees are diverse, thereby minimizing the likelihood of overfitting to specific features (Wang et al., 2021; Campagner et al., 2023). Each DT is trained independently, allowing the ensemble to capture various aspects of the data and lessen overfitting risk (Campagner et al., 2023). The final prediction is determined through a voting method in which the individual tree predictions are aggregated. The class with the maximum votes is the final predicted class.
G. AdaBoost (AB): AdaBoost, an acronym for Adaptive Boosting, is an ensemble learning algorithm in which a robust classifier is constructed by combining several weak learners. The primary aim of the AB algorithm is to train weak classifiers iteratively on different subsets of the data and allocate high weights to instances that have been incorrectly classified during each iteration (Ying et al., 2013). The final model integrates the predictions of all weak learners with varying weights, giving preference to those that perform adequately on training data. AB classifier initializes each data point in the training set with equal weights (Ding et al., 2022). In subsequent iterations, the weights are modified to focus on instances that are difficult to correctly classify. Weak learners are trained sequentially; and at each iteration, a new weak classifier is fitted to the data (Ying et al., 2013; Ding et al., 2022). Instances that are misclassified are assigned greater weights, resulting in the final model being a weighted sum of all weak classifiers. The weights are calculated based on each classifier's accuracy on the training data, with models that demonstrate higher performance contributing proportionally more to the final prediction (Haixiang et al., 2016). The final model is a combination of all weak classifiers, with the weights chosen based on their accuracy on the training data.
H. Principal Component Analysis with Logistic Regression (PC-LR): PC-LR is a method that combines the Principal Component Analysis (PCA) method (meant for dimensionality reduction) with the logistic regression (LR) algorithm (meant for classification tasks). PCA creates a new set of uncorrelated features, known as principal components, which capture the maximum variance in the data, transforming the original features (Khan and Zubair, 2020). By employing this method, the dimension of the feature space is decreased. Logistic Regression is a widely-used classifier that effectively handles linearly separable data and calculates the likelihood of an instance belonging to a specific class (Yang, 2019). By combining the dimensionality reduction capabilities of PCA with the classification power of Logistic Regression, the PC-LR method aims to retain the most informative components while reducing overall dimensionality (Aguilera et al., 2006; Yang, 2019). The input data is first transformed into a lower-dimensional space using PCA, and then logistic regression is applied to make predictions based on the reduced feature set. The LR model is trained to determine the probability that a given instance belongs to a particular class. When generating predictions for new data, the class with the highest probability is assigned as the final prediction.
Support Vector Machine - Radial Basis Function (SVM-RBF): SVM is a supervised learning technique that seeks to identify a hyperplane in N-dimensional space (N being the number of features) that best distinguishes between data points from various classes (Siddiqui et al., 2023). The objective is to optimize the margin, defined as the distance between the hyperplane and the closest data points from each class. RBF is a widely utilized kernel function in SVM. Transforming the input space to a higher-dimensional space enables the RBF kernel to effectively capture non-linear correlations in the data (Ding et al., 2021). The transformed space enables SVM to identify a non-linear decision boundary within the original feature space. SVM-RBF searches for the hyperplane in the transformed space that effectively segregates the data points into their respective classes (Ding et al., 2021). The hyperplane is used to maximize the margin, thereby establishing a robust decision boundary. SVM allows for the existence of some misclassified data points to handle cases where a perfect separation is not possible (Siddiqui et al., 2023). The key data points that influence the decision boundary are called support vectors. The RBF kernel has a parameter called the gamma (γ), which determines the shape of the decision boundary (Valero-Carreras et al., 2021). Tuning the gamma parameter is crucial, as a small gamma may lead to underfitting, while a large gamma may lead to overfitting (Sacchet et al., 2015). After establishing the decision boundary, SVM-RBF is capable of classifying newly acquired data points by determining which side of the hyperplane they lie on.
J. Perceptron (PC): The Perceptron classifier, originally developed for binary classification, can be tweaked to perform multi-class classification using a strategy called the One-vs-All and One-vs-Rest (Raju et al., 2021). The OvA strategy involves training multiple Perceptrons, each dedicated to distinguishing one specific class from all the others (Kleyko et al., 2023). For K classes, K Perceptrons are trained, where each Perceptron focusses in recognizing one class and considers the instances of that class as the positive class and all other instances as the negative class. For a multi-class problem with K classes, K Perceptrons are trained. Each Perceptron is allocated to one class, and it aims to correctly classify instances belonging to that class against instances from all other classes (Chaudhuri and Bhattacharya, 2000; Raju et al., 2021). Throughout the training phase, the weights of each Perceptron are tweaked based on the instances belonging to its allocated class. The aim is to locate weights that reduce the classification error for that specific class. During the prediction phase, each Perceptron independently predicts for a given input instance. The class associated with the Perceptron that outputs the highest confidence (largest net input) is then assigned as the predicted class for that instance (Chaudhuri and Bhattacharya, 2000; Kleyko et al., 2023). In essence, the decision for multi-class classification is made by employing a one-vs-rest strategy, where each Perceptron is treated as a binary classifier for its assigned class vs. all other classes.
K. MultiLayer Perceptron (ML-PC): This classifier is a type of artificial neural network that is devised to handle challenging ML tasks, such as multi-class classification (Chaudhuri and Bhattacharya, 2000). An ML-PC is comprised of multiple interconnected layers, such as an input layer, one/more hidden layers, and an output layer (Liu et al., 2023). Data flows through the network in a feedforward manner, with each node in a layer processing information from the previous layer and passing it to the next layer. Each node's weighted sum of inputs is subjected to activation functions such as hyperbolic tangent (tanh), sigmoid, and rectified linear unit (ReLU). These functions add non-linearity, allowing the network to understand intricate connections. The input layer represents the features of the input data, with each node corresponding to a feature and the values being the feature values (Chaudhuri and Bhattacharya, 2000). Hidden layers process information from the input layer, capturing complex, non-linear patterns in the data. The output layer generates the final predictions for each class in a multi-class setting, with each node's output representing the model's confidence in predicting that class. Backpropagation, a supervised learning technique, operates to reduce errors by iteratively modifying weights. The difference between the predicted and actual outputs is determined by the loss function. In order to minimize this difference, optimization algorithms like gradient descent are implemented, which modify the weights (Chaudhuri and Bhattacharya, 2000; Liu et al., 2023). The learning rate determines the size of each weight update.
L. Elastic Nets (EN): Elastic Nets, a regularization technique, were originally designed for linear models. In multi-class classification setups, the linear model is extended to handle multiple classes (Mol et al., 2009). Elastic Networks employ a combination of L1 (Lasso) and L2 (Ridge) methods of regularization. L1 regularization encourages sparsity in the model, promoting feature selection, while L2 regularization prevents large coefficients (Zhan et al., 2023). For multi-class classification, Elastic Nets can be applied to extend linear models to predict probabilities for multiple classes. Elastic Nets can be used in conjunction with strategies like One-vs-All (OvA) and One-vs-One (OvO) to handle multi-class challenges (Chen et al., 2018). OvA trains a distinct model for each class against the rest, whereas OvO trains models for each pair of classes. The hyperparameters alpha and l1_ratio, which control the balance between L1 and L2 regularization, must be carefully chosen in Elastic Nets. In general, the output layer utilizes a softmax activation function to transform raw model outputs into class probabilities, with the certainty that the sum of the predicted probabilities equals 1. During training, Elastic Nets optimize the model's weights using algorithms like gradient descent, to reduce the disparity between predicted and actual class probabilities (Aqeel et al., 2023). The cross-entropy loss function is frequently employed in multi-class classification tasks.

#### 2.5.2 Feature selection

Feature selection is a significant step while preparing data for ML modeling. A subset of the most pertinent features is chosen from the original set. The primary aim is to enhance model performance, simplify the model, and mitigate the risk of overfitting (Pudjihartono et al., 2022). A model with fewer features is often more interpretable, making it easier to understand the relationships between variables (Barnes et al., 2023).

There are 3 types of feature selection strategies i.e., filter, wrapper, and embedded. In this study, we employed the wrapper method (Dokeroglu et al., 2022; Pudjihartono et al., 2022; Kanyongo and Ezugwu, 2023). Wrapper methods involve evaluating the performance of a ML model based on different subsets of features. Unlike filter methods that assess feature relevance independently of the model, wrapper methods use the actual performance of the model as a criterion for selecting features. This involves training the model multiple times, which can be computationally expensive but may yield more accurate results (Kanyongo and Ezugwu, 2023). Furthermore, there are 3 types of wrapper methods viz. forward selection, recursive feature elimination, and backward elimination. In this study, we employed the step forward feature selection method. It is a specific wrapper method that builds the feature set incrementally. It starts with an empty set of features and adds them one at a time, based on how they affect the model's performance. The process involves five steps, illustrated in Figure 3.

#### 2.5.3 Cross-validation and hyperparameter optimization

The objective of this study was to construct such a model that can exhibit optimal generalized performance rather than only for the cases used during training. Consequently, cross-validation gives an estimate of the overall performance for each hyperparameter configuration (Khan and Zubair, 2022a). To achieve this, the train data was divided into 5 folds. Instances from each fold were held-out from the training process, while the remaining cases were trained iteratively. Subsequently, the algorithm was then applied to the held-out samples following their training.

In this study, ten iterations of a 5-fold repeated stratified CV training and testing approach were implemented to maintain the distribution of classes across each fold (Figure 4). The Scikit-Learn library in Python, for instance, provides a RepeatedStratifiedKFold class that was employed for implementing this type of cross-validation. This was employed for evaluating and fine-tuning the models since we were dealing with scenarios where data variability and class imbalance challenges needed to be carefully managed.

An imbalanced classification problem can pose a significant challenge when building a ML model, especially when the data distribution is skewed toward the target variable (Kanyongo and Ezugwu, 2023). In such cases, if not addressed properly, the model may perform poorly, resulting in low accuracy. In the present study, we aimed to resolve the imbalanced classification problem by employing the Synthetic Minority Oversampling Technique (SMOTE) method. SMOTE is a data augmentation technique, designed to handle minority classes (Kohavi, 1995; Chawla et al., 2022). As previously stated, there were 3 classes of the target variable, including cognitively normal, MCI, and AD subjects. But we discovered a significant disparity in the MCI and AD classes. As a result, the built ML model resulted in poor performance and low accuracy. To address this issue, we utilized SMOTE analysis to oversample the minority class, which balanced the class distribution without adding any further information to the ML model.

The hyperparameters were fine-tuned before assessing each classifier. Hyperparameters are essential for structuring ML models and are not learned from the data during training. Hyperparameter optimization is the process of systematically searching for the best combination of hyperparameter values to achieve optimal performance from the model (Yang and Shami, 2020; Khan and Zubair, 2022a). It is crucial to optimize these hyperparameters to obtain the best possible results when applied to unseen instances. ML algorithms often have one or more hyperparameters that can be adjusted during the training process. By varying these hyperparameters, the algorithm's prediction performance can be varied. In this study, each model was trained using specific hyperparameter configurations to optimize the hyperparameters for each employed ML algorithm.

### 2.6 Analyze results

The purpose of this step was to assess the performance of the base models and determine whether they exhibited improved performance. If not, the model was subjected to additional training for an extended period of time, using a varied set of optimization hyperparameters.

### 2.7 Evaluate test performance

This stage determined the performance of test splits on the test data. If the performance was found to be poor, the test distribution was reevaluated, and any discrepancies were rectified by creating equitable splits. If the performance proved to be acceptable (with a high level of accuracy), the subsequent step was carried out and a meta-model was constructed.

### 2.8 Build meta-model

To construct a meta-model, we employed an ensemble learning approach that involved selecting different outputs generated by 12 machine learning classifiers after modeling and optimization. These classifiers were made to run in parallel and subjected to a voting and stacking process. Majority voting and majority stacking are ensemble learning approaches that integrate the predictions of numerous independent models to improve overall predictive performance (Raza, 2019; Dolo and Mnkandla, 2023). Both methods involve aggregating the decisions of multiple models, but they differ in their approaches. Majority voting encompasses multiple models making independent predictions on a given input, with the final prediction being determined by the majority vote or consensus of these individual predictions (Zhao et al., 2023). On the other hand, majority stacking is a more sophisticated method that trains a meta-model, often referred to as a stacker or meta-learner, to combine the predictions of multiple base models (Aboneh et al., 2022; Dolo and Mnkandla, 2023). The process of averaging predictions involves selecting the class with the most votes (the statistical mode) or the class with the highest summed probability. Stacking extends this method by enabling any machine learning model to learn how to integrate predictions from contributing members optimally.

After applying majority voting and majority stacking, we evaluated their performances individually and selected the approach that resulted in the best overall performance. The selected model, whether it was based on majority voting or majority stacking, effectively served as a meta-model in our ensemble learning approach. This approach often results in improved generalization and performance compared to relying on a single model.

### 2.9 Generalized performance metrics: evaluate and compare

**Figure 5:**
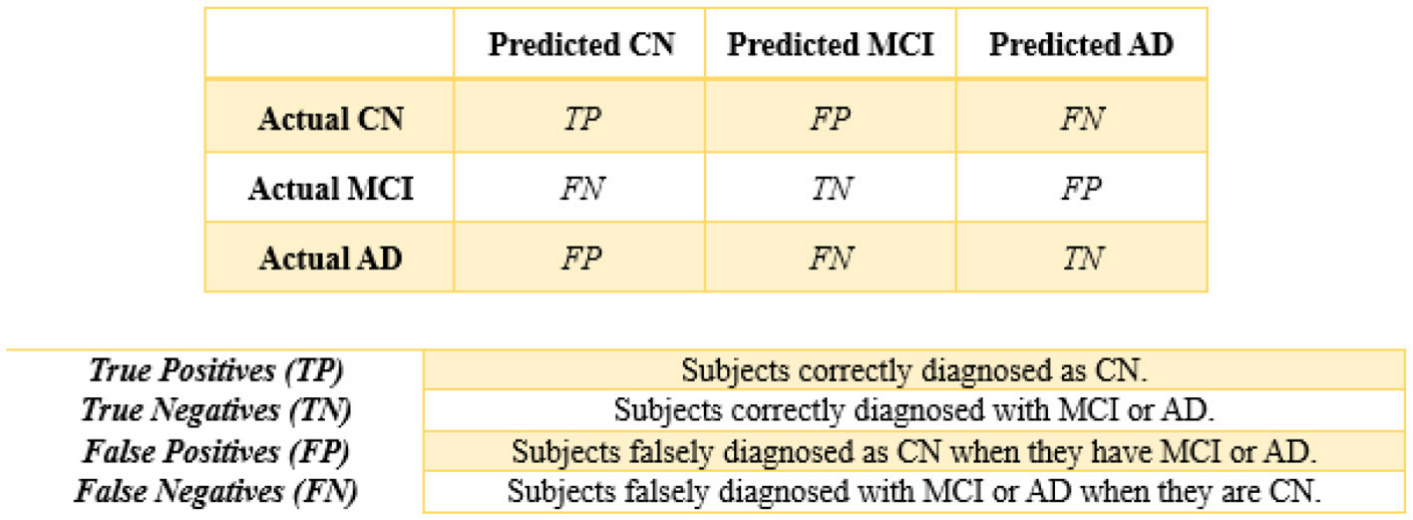
3 × 3 confusion matrix.

Finally, performance evaluations were conducted and compared to all applied approaches for each of the four controllers established in this study. We employed accuracy, precision, recall, and F1-score as classification performance metrics for both the base model and meta-model. The meta-model was evaluated using two metrics for classification error: Hamming loss and the Jaccard index. Each parameter was determined in this study by employing a 3 × 3 confusion matrix (Figure 5). In ML classification, the confusion matrix is a widely used method that evaluates the performance of a model through a comparison between its predictions to the actual labels in a specific dataset. Table 5 presents a detailed description of each of the metrics derived from the confusion matrix.

**Table 5:** Description of the measures used.

**Metric**	**Definition**	**Formula**
Accuracy	- Accuracy is defined as the ratio of correctly predicted occurrences to total occurrences. - It measures the overall correctness of the predictions of the model.	(TP + TN)/(TP + TN + FP + FN)
Precision	- Precision is the proportion of correctly predicted positive occurrences to total predicted positives.	TP/(TP + FN)
Recall	- It represents the proportion of accurately predicted positive outcomes to the total number of actual positives.	TP/(TP + FN)
F1-score	- The F1-score is the harmonic mean of precision and recall. - It is a single metric that balances precision and recall.	2 ^*^ (Precision ^*^ Recall)
Hamming loss	- It calculates the proportion of incorrectly predicted labels. - In multi-label classification, where each instance may belong to multiple categories, Hamming Loss calculates the proportion of labels that are incorrectly predicted.	(FP + FN)/(Total Classes ^*^ Total Instances)
Jaccard index	- The Jaccard Index quantifies the degree of similarity that exists between the predicted and actual sets of labels. - It measures the intersection over the union of the predicted and actual sets of labels, indicating how closely the predicted labels match the true labels.	TP/(TP + FP + FN)

### 2.10 Development environment and base settings of classification algorithms

To ensure the replicability of our study, we documented the development environment, including software and hardware specifications, as well as the configuration settings of the classification algorithms employed. The implementation of our predictive models for the diagnosis of AD pathologies was conducted using the Anaconda distribution of Python, which provides a comprehensive environment for data science and ML tasks. Anaconda includes a wide range of pre-installed libraries and tools, making it well-suited for developing and deploying ML models.

Jupyter Notebook, a web-based interactive computing environment, was utilized for the implementation of the study. Jupyter Notebooks offer a convenient interface for writing code, executing experiments, visualizing results, and cohesively presenting findings. The hardware utilized for model training and evaluation consisted of a system with an Intel Core-6100U CPU running at a base frequency of 2.30 GHz and 4 GB of RAM.

The classification algorithms utilized in our study were configured with base settings to achieve optimal performance. The base settings of these algorithms were carefully selected and fine-tuned to balance model complexity and predictive performance. Hyperparameters were optimized using techniques like grid search and random search. For MLR, the base settings included Cs (regularization strength) set to 10, cv (cross-validation) set to 5, and multi-class set to “multinomial.” For KNN, the base settings included n_neighbors set to 5, weights set to “uniform,” and algorithm set to “auto,” with optimization focused on the number of neighbors.

LDA used the default settings provided by the “svd” (Singular Value Decomposition) solver, while QDA utilized a regularization parameter of 0.0 and did not require specific hyperparameter optimization. Decision Trees were optimized for parameters like max_depth (up to 20), cv set to 5, and n_jobs set to 2 respectively. Furthermore, for Random Forest, the settings consisted of n_estimators set to 50, max_depth set to 20, cv set to 5, and n_jobs set to 4, with optimization performed for the number of estimators and maximum depth. AdaBoost was optimized for parameters like max_depth (up to 20) and learning_rate (0.05, 0.1, 0.5), with a base estimator as Decision Tree.

Utilizing Cs set to 10, cv set to 5, and multi_class set to “ovr” as base settings for logistic regression, with default settings used for PCA for hyperparameter optimization in the PC-LR algorithm. SVM-RBF used parameters like C (ranging from 0.001 to 1,000), gamma (values ranging from 0 to 1), kernel set to “rbf,” and decision_function_shape set to “ovr” for optimization. Perceptron was optimized for the alpha parameter (ranging from 0.0001 to 10,000) and the penalty set to “l2,” with optimization centered around the alpha parameter. Multi-layer Perceptron employed settings for parameters such as hidden_layer_sizes set to 100, activation function set to “relu,” and learning_rate set to “constant.” Elastic Nets were configured with base settings such as l1_ratio (ranging from 0.1 to 1) and alphas (values ranging from 0 to 10). Also, pre-processing steps were applied uniformly to ensure consistency in model training and evaluation.

## 3 Results

This section presents the findings of the hybrid-clinical ML modeling for each of the individual set controllers. Initially, the base models are discussed, followed by the meta-model.

### 3.1 For baseline (C = 1)

Table 6 presents the modeling results for the base ML models. These results are for all three approaches to handling missing data, as discussed in Section 2.3.1. The feature selection process identified the following features to be included: Age, Education, Gender, CDR_SB, ADAS13, ADASQ4, MMSE, RAVLT-I, RAVLT-L, RAVLT-PF, TRABSCOR, LDETOTAL, FAQ, mPACCdigit, Ventricles, WholeBrain, Hippocampus, Entorhinal, Fusiform, ICV, MidTemp, Ethnicity, Race category, Married status, and APOE4. Moreover, Table 7 summarizes the outcome of the constructed Meta-Model as well as the metrics used to evaluate performance.

**Table 6:** Performance results on Base models (C=1).

	**Without imputation**					**Imputation with mean**					**Model imputation**				
**Base model**	**Train Acc. (%)**	**Test Acc. (%)**	**Pr. (%)**	**Rec. (%)**	**F1-sc**.	**Train Acc. (%)**	**Test Acc. (%)**	**Pr. (%)**	**Rec. (%)**	**F1-sc**.	**Train Acc. (%)**	**Test Acc. (%)**	**Pr. (%)**	**Rec. (%)**	**F1-sc**.
LR	95.80	92.80	93.00	93.00	0.93	95.60	94.80	95.00	95.00	0.95	95.60	95.30	95.00	95.00	0.95
KNN	89.60	89.20	90.00	90.00	0.89	90.50	87.70	91.00	91.00	0.90	90.00	89.50	90.00	90.00	0.89
LDA	95.30	94.60	95.00	95.00	0.95	93.80	93.30	94.00	94.00	0.94	93.40	93..10	94.00	93.00	0.93
QDA	82.70	77.10	77.00	77.00	0.76	54.90	54.50	69.00	55.00	0.45	66.80	65.00	66.00	65.00	0.64
DT	95.30	94.60	95.00	95.00	0.95	94.40	93.80	94.00	94.00	0.94	94.40	93.80	94.00	94.00	0.94
RF	98.90	94.00	94.00	94.00	0.94	99.80	95.26	96.00	96.00	0.96	99.80	95.30	95.00	95.00	0.95
AB	96.70	95.20	95.00	95.00	0.95	94.20	93.80	94.00	94.00	0.94	94.20	93.80	94.00	94.00	0.94
PCA-LR	92.20	91.00	91.00	91.00	0.91	92.30	89.10	89.00	89.00	0.89	91.60	90.50	91.00	91.00	0.91
SVM-RBF	100.00	92.20	92.00	92.00	0.92	95.60	91.50	91.00	91.00	0.91	95.70	91.50	92.00	91.00	0.91
PC	92.80	89.30	93.00	93.00	0.93	89.60	86.50	90.00	90.00	0.90	89.60	88.50	90.00	90.00	0.90
ML-PC	61.30	58.00	40.00	58.00	0.47	72.00	71.10	56.00	71.00	0.62	70.00	70.60	56.00	71.00	0.62
EN	92.80	92.00	94.00	93.00	0.93	92.40	91.90	93.00	92.00	0.93	92.00	91.80	92.00	92.00	0.92

**Table 7:** Performance result: meta-model (C = 1).

	**Without imputation**		**Imputation with mean**		**Model imputation**	
	**Train Acc. (%)**	**Test Acc. (%)**	**Train Acc. (%)**	**Test Acc. (%)**	**Train Acc. (%)**	**Test Acc. (%)**
Accuracy	97.33	95.78	96.00	95.70	96.22	95.73
Precision (%)	95.88		95.35		95.84	
Recall (%)	95.78		95.26		95.73	
F-measure	0.96		0.95		0.96	
Hamming loss	0.04		0.04		0.04	
Jaccard index	0.92		0.91		0.92	

As shown in Table 6, the AdaBoost algorithm without imputation, Random Forest with mean imputation, Logistic Regression (multinomial), and Random Forest with model imputation techniques all achieved a higher accuracy of 95.20%, 95.26%, and 95.30% on the test set, respectively. Subsequently, as a Meta-Model (Table 7), the improved accuracy was demonstrated using a no-imputation approach with a 95.78% accuracy on the test set. On the test set, mean imputation achieved 95.70% accuracy, whereas model imputation achieved 95.73% accuracy. Additionally, the performance of the Meta-Model with the no-imputation technique was determined to be the best among all, as it gave an accuracy of ~96.0%, a precision value of ~96.0%, a recall value of ~96.0%, and F1-score of 0.96, which was close to 1.0. A high F-measure, close to 1.0, is considered the best measure. Furthermore, the classification error i.e., Hamming Loss, gave a value of 0.04, which was close to 0, considered the best value. Finally, the Jaccard Index value of 0.92, near 1.0, suggested that the created Meta-Model reflected the best classification.

### 3.2 For baseline + drug (C = 2)

Table 8 shows the modeling results for the base ML models. The feature selection process identified the following features to be included for controller 2 (baseline + drug): Age, Education, Gender, CDR-SB, MMSE, RAVLT-I, RAVLT-L, RAVLT-F, LDETOTAL, TRABSCOR, DIGITSCOR, Ventricles, WholeBrain, Hippocampus, Fusiform, MidTemp, Ethnicity, Race category, Married status, Blood thinner, Calcium, Cholesterol, Cognitive, and Vitamin D. Additionally, Table 9 presents the findings of the built Meta-Model as well as the metrics used to evaluate performance.

**Table 8:** Outcome-based on base models (C = 2).

	**Without imputation**					**Imputation with mean**					**Model imputation**				
**Base model**	**Train Acc. (%)**	**Test Acc. (%)**	**Pr. (%)**	**Rec. (%)**	**F1-sc**.	**Train Acc. (%)**	**Test Acc. (%)**	**Pr. (%)**	**Rec. (%)**	**F1-sc**.	**Train Acc. (%)**	**Test Acc. (%)**	**Pr. (%)**	**Rec. (%)**	**F1-sc**.
LR	96.00	92.20	92.00	92.00	0.92	96.00	92.20	92.00	92.00	0.92	96.00	92.20	92.00	92.00	0.92
KNN	90.40	88.40	90.00	90.00	0.90	88.40	90.40	90.00	90.00	0.90	90.40	88.40	90.00	90.00	0.90
LDA	95.30	94.00	94.00	94.00	0.94	95.30	94.00	94.00	94.00	0.94	95.30	94.00	94.00	94.00	0.94
QDA	53.10	48.80	60.00	49.00	0.42	53.10	48.80	60.00	49.00	0.42	53.10	48.80	60.00	49.00	0.42
DT	96.70	95.20	95.00	95.00	0.95	95.80	95.10	96.00	96.00	0.96	95.80	95.10	96.00	96.00	0.96
RF	99.80	93.40	93.00	93.00	0.93	100.00	94.60	95.00	95.00	0.94	99.60	95.20	95.00	95.00	0.95
AB	96.50	94.00	93.87	94.00	0.93	96.50	96.18	94.56	94.58	0.94	96.40	95.00	95.15	95.18	0.95
PCA-LR	92.20	91.60	92.00	92.00	0.92	92.20	91.60	92.00	92.00	0.92	92.20	91.60	92.00	92.00	0.92
SVM-RBF	96.00	94.60	94.00	95.00	0.94	96.00	94.60	94.00	95.00	0.94	95.80	94.60	94.00	95.00	0.94
PC	90.00	85.50	87.00	86.00	0.86	90.00	85.50	87.00	86.00	0.86	90.00	88.50	87.00	86.00	0.86
ML-PC	57.30	58.40	43.00	58.00	0.47	60.00	58.40	40.00	58.00	0.47	71.10	68.20	62.00	71.00	0.65
EN	94.20	92.20	94.00	92.00	0.93	94.20	92.20	94.00	92.00	0.93	94.20	92.20	94.00	92.00	0.93

**Table 9:** Result: meta-model (C = 2).

	**Without imputation**		**Imputation with mean**		**Model imputation**	
	**Train Acc. (%)**	**Test Acc. (%)**	**Train Acc. (%)**	**Test Acc. (%)**	**Train Acc. (%)**	**Test Acc. (%)**
Accuracy	97.00	96.40	96.44	96.40	97.00	96.40
Precision (%)	96.00		96.00		96.00	
Recall (%)	96.00		96.00		96.00	
F-measure	0.96		0.96		0.96	
Hamming loss	0.06		0.05		0.04	
Jaccard index	0.89		0.89		0.91	

As shown in Table 8, the AdaBoost algorithm produced an accuracy of 94.00% on the test set when used without imputation, 96.18% with mean imputation, and 95.00% with model imputation. Following that, using the no-imputation technique with a 96.40% accuracy on the test set, the improved accuracy was demonstrated using a Meta-Model. On the test set, an improved accuracy of 96.40% was achieved for all three techniques. When compared to the base model, it was established that the performance of the Meta-Model was way better. Other performance metrics including precision, recall, and F-score demonstrated a performance improvement. Hamming loss was calculated and yielded a value of 0.06, 0.05, and 0.04 respectively, which are regarded to be as acceptable. Additionally, the Jaccard Index values of 0.89, 0.89, and 0.91, which were all near 1.0, indicated that the Meta-Model built was accurate.

Following that, we present the results for all of the features above (excluding the drug list), and only one drug at a time was examined. Table 10 summarizes the modeling results for base models, whereas Table 11 summarizes the Meta-Model results when just one particular drug was considered in addition to baseline characteristics.

**Table 10:** Base model: baseline features + individual drug.

	**Baseline** + **blood thinner drug**														
	**Without imputation**					**Imputation with mean**					**Model imputation**				
**Base model**	**Train Acc. (%)**	**Test Acc. (%)**	**Pr. (%)**	**Rec. (%)**	**F1-sc**.	**Train Acc. (%)**	**Test Acc. (%)**	**Pr. (%)**	**Rec. (%)**	**F1-sc**.	**Train Acc. (%)**	**Test Acc. (%)**	**Pr. (%)**	**Rec. (%)**	**F1-sc**.
LR	94.70	94.00	94.00	94.00	0.94	94.70	94.00	94.00	94.00	0.94	94.70	94.00	94.00	94.00	0.94
KNN	93.40	91.80	93.00	93.00	0.93	93.40	91.80	93.00	93.00	0.93	93.40	91.80	93.00	93.00	0.93
LDA	93.00	92.80	93.00	93.00	0.93	93.00	92.80	93.00	93.00	0.93	93.00	92.80	93.00	93.00	0.93
QDA	20.00	17.00	03.00	17.0	0.05	20.00	17.00	03.00	17.0	0.05	20.00	17.00	03.00	17.0	0.05
DT	97.30	95.20	95.00	95.00	0.95	98.40	95.20	95.00	95.00	0.95	98.00	95.20	95.00	95.00	0.95
RF	100.00	95.20	95.00	95.00	0.95	99.10	94.60	94.00	95.00	0.94	99.60	95.20	95.00	95.00	0.95
AB	95.78	93.37	93.35	93.38	0.93	95.78	93.37	93.35	93.38	0.93	98.00	95.80	96.00	96.00	0.96
PCA-LR	92.20	90.20	92.00	92.00	0.92	92.20	90.20	92.00	92.00	0.92	92.20	90.20	92.00	92.00	0.92
SVM-RBF	97.30	94.00	94.00	94.00	0.94	97.30	94.00	94.00	94.00	0.94	97.30	94.00	94.00	94.00	0.94
PC	92.80	87.10	93.00	93.00	0.92	92.80	87.10	93.00	93.00	0.92	92.80	87.10	93.00	93.00	0.92
ML-PC	78.30	73.10	66.00	78.00	71.00	78.30	73.30	67.00	78.00	72.00	78.00	71.30	66.00	78.00	71.00
EN	92.70	92.20	93.00	92.00	0.93	92.70	92.20	93.00	92.00	0.93	92.70	92.20	93.00	92.00	0.93
**Baseline** + **calcium drug**															
**Base model**	**Train Acc. (%)**	**Test Acc. (%)**	**Pr. (%)**	**Rec. (%)**	**F1-sc**.	**Train Acc. (%)**	**Test Acc. (%)**	**Pr. (%)**	**Rec. (%)**	**F1-sc**.	**Train Acc. (%)**	**Test Acc. (%)**	**Pr. (%)**	**Rec. (%)**	**F1-sc**.
LR	95.00	94.60	95.00	95.00	0.95	95.00	94.60	95.00	95.00	0.95	95.00	94.60	95.00	95.00	0.95
KNN	93.40	92.00	93.00	93.00	0.93	93.40	92.00	93.00	93.00	0.93	93.40	92.00	93.00	93.00	0.93
LDA	93.10	92.20	92.00	92.00	0.92	93.10	92.20	92.00	92.00	0.92	93.10	92.20	92.00	92.00	0.92
QDA	33.10	31.60	11.00	33.00	0.16	33.10	31.60	11.00	33.00	0.16	33.10	31.60	11.00	33.00	0.16
DT	99.00	95.00	95.00	95.00	0.95	98.20	95.20	95.00	95.00	0.95	98.20	94.60	95.00	95.00	0.95
RF	100.00	94.00	94.00	94.00	0.94	99.10	95.20	95.00	95.00	0.95	98.20	94.80	96.00	96.00	0.96
AB	95.56	93.37	93.53	93.37	0.93	96.50	95.00	95.00	95.00	0.95	96.50	94.40	94.00	94.00	0.94
PCA-LR	92.20	90.20	92.00	92.00	0.92	92.20	90.20	92.00	92.00	0.92	92.20	90.20	92.00	92.00	0.92
SVM-RBF	94.20	92.80	93.00	93.00	0.93	94.20	92.80	93.00	93.00	0.93	94.20	92.80	93.00	93.00	0.93
PC	91.00	89.00	92.00	91.00	0.91	91.00	88.70	92.00	91.00	0.91	91.00	88.70	92.00	91.00	0.91
ML-PC	77.10	73.00	65.00	77.00	0.70	80.70	74.20	68.00	81.00	0.74	79.00	75.10	67.00	79.00	0.72
EN	92.40	92.20	93.00	92.00	0.93	92.40	92.20	93.00	92.00	0.93	92.40	92.20	93.00	92.00	0.93
**Baseline** + **cholesterol drug**															
**Base model**	**Train Acc. (%)**	**Test Acc. (%)**	**Pr. (%)**	**Rec. (%)**	**F1-sc**.	**Train Acc. (%)**	**Test Acc. (%)**	**Pr. (%)**	**Rec. (%)**	**F1-sc**.	**Train Acc. (%)**	**Test Acc. (%)**	**Pr. (%)**	**Rec. (%)**	**F1-sc**.
LR	95.20	94.70	95.00	95.00	0.95	95.20	94.70	95.00	95.00	0.95	95.20	94.70	95.00	95.00	0.95
KNN	93.60	93.40	93.00	93.00	0.93	93.60	93.40	93.00	93.00	0.93	93.60	93.40	93.00	93.00	0.93
LDA	93.10	92.80	93.00	93.00	0.93	93.10	92.80	93.00	93.00	0.93	93.10	92.80	93.00	93.00	0.93
QDA	20.00	17.00	03.00	17.0	0.05	20.00	17.00	03.00	17.0	0.05	20.00	17.00	03.00	17.0	0.05
DT	95.80	95.10	96.00	96.00	0.96	98.00	94.60	95.00	95.00	0.95	99.60	94.60	95.00	95.00	0.95
RF	98.40	95.80	96.00	96.00	0.96	99.00	93.40	93.00	93.00	0.93	96.22	94.00	94.00	94.00	0.94
AB	95.56	94.00	94.00	94.00	0.94	96.22	96.02	94.00	94.00	0.94	98.00	95.80	96.00	96.00	0.96
PCA-LR	92.20	90.20	92.00	92.00	0.92	92.20	90.20	92.00	92.00	0.92	92.20	90.20	92.00	92.00	0.92
SVM-RBF	97.30	94.00	94.00	94.00	0.94	97.30	94.00	94.00	94.00	0.94	97.30	94.00	94.00	94.00	0.94
PC	92.20	91.80	93.00	92.00	0.92	92.20	91.80	93.00	92.00	0.92	92.20	91.80	93.00	92.00	0.92
ML-PC	80.10	71.60	69.00	80.00	73.00	60.80	60.70	44.00	61.00	0.50	73.30	77.70	65.00	78.00	0.71
EN	92.40	92.20	93.00	92.00	0.93	92.40	92.20	93.00	92.00	0.93	92.40	92.20	93.00	92.00	0.93
**Baseline** + **cognitive drug**															
**Base model**	**Train Acc. (%)**	**Test Acc. (%)**	**Pr. (%)**	**Rec. (%)**	**F1-sc**.	**Train Acc. (%)**	**Test Acc. (%)**	**Pr. (%)**	**Rec. (%)**	**F1-sc**.	**Train Acc. (%)**	**Test Acc. (%)**	**Pr. (%)**	**Rec. (%)**	**F1-sc**.
LR	94.70	94.60	95.00	95.00	0.95	94.70	94.60	95.00	95.00	0.95	94.70	94.60	95.00	95.00	0.95
KNN	93.80	93.40	93.00	93.00	0.93	93.80	93.40	93.00	93.00	0.93	93.80	93.40	93.00	93.00	0.93
LDA	93.00	92.20	92.00	92.00	0.92	93.00	92.20	92.00	92.00	0.92	93.00	92.20	92.00	92.00	0.92
QDA	33.10	31.60	11.00	33.00	0.16	33.10	31.60	11.00	33.00	0.16	33.10	31.60	11.00	33.00	0.16
DT	97.30	95.80	96.00	96.00	0.96	95.78	93.37	93.35	93.37	0.93	95.11	93.37	93.35	93.37	0.93
RF	99.30	96.00	96.00	96.00	0.96	99.80	93.37	93.35	93.37	0.93	95.80	92.20	92.00	92.00	0.92
AB	96.41	96.00	96.00	96.00	0.96	98.00	93.37	93.35	93.37	0.93	98.00	95.50	96.00	96.00	0.96
PCA-LR	92.20	90.20	92.00	92.00	0.92	92.20	90.20	92.00	92.00	0.92	92.20	90.20	92.00	92.00	0.92
SVM-RBF	94.40	92.80	93.00	93.00	0.93	94.40	92.80	93.00	93.00	0.93	94.40	92.80	93.00	93.00	0.93
PC	86.10	85.10	87.00	86.00	0.86	86.10	85.10	87.00	86.00	0.86	86.10	85.10	87.00	86.00	0.86
ML-PC	76.50	76.50	65.00	77.00	0.70	77.70	73.60	66.00	78.00	0.71	80.10	75.30	68.00	80.00	0.73
EN	92.70	91.60	93.00	92.00	0.92	92.70	91.60	93.00	92.00	0.92	92.70	91.60	93.00	92.00	0.92
**Baseline** + **vitamin D drug**															
**Base model**	**Train Acc. (%)**	**Test Acc. (%)**	**Pr. (%)**	**Rec. (%)**	**F1-sc**.	**Train Acc. (%)**	**Test Acc. (%)**	**Pr. (%)**	**Rec. (%)**	**F1-sc**.	**Train Acc. (%)**	**Test Acc. (%)**	**Pr. (%)**	**Rec. (%)**	**F1-sc**.
LR	95.20	94.70	95.00	95.00	0.95	95.20	94.70	95.00	95.00	0.95	95.20	94.70	95.00	95.00	0.95
KNN	93.40	91.60	93.00	93.00	0.93	93.40	91.60	93.00	93.00	0.93	93.40	91.60	93.00	93.00	0.93
LDA	93.00	92.80	93.00	93.00	0.93	93.00	92.80	93.00	93.00	0.93	93.00	92.80	93.00	93.00	0.93
QDA	33.10	31.60	11.00	33.00	0.16	33.10	31.60	11.00	33.00	0.16	33.10	31.60	11.00	33.00	0.16
DT	96.00	95.10	96.00	96.00	0.96	98.40	95.20	95.00	95.00	0.95	96.00	94.10	94.00	94.00	0.94
RF	100.00	95.00	93.35	93.38	0.93	98.20	95.20	95.00	95.00	0.95	97.30	94.00	94.00	94.00	0.94
AB	98.00	95.00	93.35	93.38	0.93	96.00	94.00	93.47	94.00	0.94	98.00	94.80	94.04	93.38	0.93
PCA-LR	92.20	90.20	92.00	92.00	0.92	92.20	90.20	92.00	92.00	0.92	92.20	90.20	92.00	92.00	0.92
SVM-RBF	97.30	94.0	94.00	94.00	0.94	97.30	94.0	94.00	94.00	0.94	97.30	94.0	94.00	94.00	0.94
PC	84.0	87.30	90.00	87.00	0.86	84.0	87.30	90.00	87.00	0.86	84.0	87.30	90.00	87.00	0.86
ML-PC	78.30	73.30	66.00	78.00	0.71	77.10	70.00	65.00	77.00	70.00	85.50	80.00	88.00	86.00	0.85
EN	93.00	92.20	93.00	92.00	0.93	93.00	92.20	93.00	92.00	0.93	93.00	92.20	93.00	92.00	0.93

**Table 11:** Meta-model: baseline features + individual drug.

	**Without imputation**		**Imputation with mean**		**Model imputation**	
	**Train Acc. (%)**	**Test Acc. (%)**	**Train Acc. (%)**	**Test Acc. (%)**	**Train Acc. (%)**	**Test Acc. (%)**
**Baseline** + **blood thinner drug**						
Accuracy	98.00	95.80	96.50	96.40	96.22	96.00
Precision (%)	96.00		96.00		94.04	
Recall (%)	96.00		96.00		94.00	
F-measure	0.96		0.96		0.94	
Hamming loss	0.06		0.06		0.06	
Jaccard index	0.90		0.90		0.89	
**Baseline** + **calcium drug**						
Accuracy	96.50	96.40	96.18	96.00	95.50	95.00
Precision (%)	96.00		94.01		94.01	
Recall (%)	96.00		94.00		94.00	
F-measure	0.96		0.94		0.94	
Hamming loss	0.06		0.06		0.06	
Jaccard index	0.88		0.89		0.89	
**Baseline** + **cholesterol drug**						
Accuracy	96.40	96.00	96.41	96.40	97.10	96.40
Precision (%)	96.00		96.00		96.00	
Recall (%)	96.00		96.00		96.00	
F-measure	0.96		0.96		0.96	
Hamming loss	0.06		0.06		0.06	
Jaccard index	0.90		0.90		0.90	
**Baseline** + **cognitive drug**						
Accuracy	96.41	96.40	97.30	95.80	97.30	95.80
Precision (%)	96.00		96.00		96.00	
Recall (%)	96.00		96.00		96.00	
F-measure	0.96		0.96		0.96	
Hamming loss	0.06		0.06		0.06	
Jaccard index	0.88		0.88		0.88	
**Baseline** + **vitamin D drug**						
Accuracy	96.00	95.80	96.41	96.40	95.56	95.00
Precision (%)	96.00		96.00		95.00	
Recall (%)	96.00		96.00		95.00	
F-measure	0.96		0.96		0.95	
Hamming loss	0.06		0.06		0.06	
Jaccard index	0.88		0.89		0.88	

As shown in Table 10, all of the algorithms studied had an accuracy between 94.00% and 96.00%. The following conclusions were drawn: Comparing Tables 8, 10 results for Base Models revealed that when only the cognitive drug was considered, the no-imputation technique (96.00%) using the AdaBoost classifier produced better accurate results. If just cholesterol-lowering supplements and baseline characteristics were considered, the accuracy of the imputation with the mean technique was 96.02%. When compared to Table 8, which included the performance results for the selected baseline features and the five medications, it was discovered that the AdaBoost classifier had an accuracy of 96.18% when using the Linear Regression imputation approach. If just blood thinners or cholesterol-lowering supplements were considered in addition to baseline characteristics, the accuracy for imputation with mean and Linear Regression techniques was 95.80% and 95.80%, respectively. When compared to Table 8, the accuracy of the AdaBoost classifier for the model imputation approach was 95.00%.

Following that, Table 11 summarizes the Meta-Model developed for all of the baseline characteristics assessed, as well as for a single drug. It revealed that the performance accuracy of the five Meta-Models was close to 96.0% in three of the scenarios (Table 11). The precision, recall, and F1-score values, as well as the classification errors, were all highly commendable as well. This indicated that the created model performed well for both if all drugs were taken into consideration or if any single drug was included for the diagnosis of the response variable as CN, AD, or MCI, which was a positive factor. When compared to the results in Table 9, this indicated that the Meta-Model yielded rather similar outcomes regardless of whether a specific drug was employed or all five medications were included. No one medication had a discernible effect on the diagnosis of AD, CN, or MCI. Each of them produced an identical result.

### 3.3 For baseline + protein (C = 3)

The features that were selected post-feature selection mechanism are Age, Education, Gender, CDR-SB, ADAS13, MMSE, RAVLT-L, LDETOTAL, DIGITSCOR, TRABSCOR, mPACCdigit, Fusiform, MidTemp, Ethnicity, Race category, Married status, Aβ, Tau, and PTau. To categorize the response variables CN, MCI, and AD based on these features, ML modeling was performed. Table 12 presents the results for the base ML models. In addition, Table 13 highlights the findings of the Meta-Model that was developed as a result of this process.

**Table 12:** Performance result on base model for C = 3.

	**Without imputation**					**Imputation with mean**					**Model imputation**				
**Base model**	**Train Acc. (%)**	**Test Acc. (%)**	**Pr. (%)**	**Rec. (%)**	**F1-sc**.	**Train Acc. (%)**	**Test Acc. (%)**	**Pr. (%)**	**Rec. (%)**	**F1-sc**.	**Train Acc. (%)**	**Test Acc. (%)**	**Pr. (%)**	**Rec. (%)**	**F1-sc**.
LR	97.10	96.40	97.00	96.00	0.96	98.20	86.00	86.00	86.00	0.86	98.20	87.30	87.00	87.00	0.87
KNN	90.00	80.00	80.00	80.00	0.80	90.00	80.40	82.00	80.00	0.80	88.70	79.00	80.00	79.00	0.78
LDA	95.00	94.00	94.00	94.00	0.94	97.20	82.10	84.00	82.00	0.82	97.20	83.00	85.00	83.00	0.83
QDA	58.20	26.20	42.00	26.00	0.15	53.50	30.40	31.00	30.00	0.18	53.50	30.40	31.00	30.00	0.18
DT	97.00	96.40	97.00	96.00	0.96	96.10	85.00	86.00	85.00	0.84	97.50	88.40	89.00	88.00	0.88
RF	99.50	96.40	96.00	96.00	0.96	100.00	86.00	88.00	88.00	0.87	100.00	85.00	87.00	85.00	0.84
AB	98.00	96.43	96.00	96.00	0.96	97.50	86.60	88.00	87.00	0.86	97.50	87.00	88.00	87.00	0.86
PCA-LR	90.00	89.30	90.00	89.00	0.89	90.10	79.00	80.00	79.00	0.78	90.50	77.00	78.00	77.00	0.77
SVM-RBF	99.50	93.00	93.00	93.00	0.93	100.00	86.00	86.00	86.00	0.86	100.00	87.00	87.00	87.00	0.87
PC	85.60	84.50	88.00	85.00	0.84	90.50	77.00	78.00	77.00	0.76	90.10	71.00	74.00	71.00	0.68
ML-PC	57.70	57.10	69.00	57.00	0.60	69.70	58.00	43.00	58.00	0.49	48.20	45.00	20.00	45.00	0.28
EN	93.30	90.50	92.00	90.00	0.91	94.40	83.00	85.00	83.00	0.83	94.00	86.00	87.00	86.00	0.86

**Table 13:** Result: meta-model (C = 3).

	**Without imputation**		**Imputation with mean**		**Model imputation**	
	**Train Acc. (%)**	**Test Acc. (%)**	**Train Acc. (%)**	**Test Acc. (%)**	**Train Acc. (%)**	**Test Acc. (%)**
Accuracy	98.08	97.60	97.54	88.40	98.30	89.00
Precision (%)	98.00		88.53		89.49	
Recall (%)	98.00		88.71		89.61	
F-measure	0.98		0.86		0.89	
Hamming loss	0.03		0.14		0.13	
Jaccard index	0.93		0.75		0.77	

Table 12 exhibited that when the AdaBoost technique was employed without imputation on the test set, it delivered a high accuracy of 96.43%. When compared to the Meta-Model, it achieved an accuracy of 97.60% for the same applied approach. The accuracy of the Base Model was 86.0% with mean imputation and 87.30% with model imputation techniques, respectively. Following that, using an imputation with mean approach with an accuracy of 88.40% and an imputation with Linear Regression technique with an accuracy of 89.00%, the enhanced accuracy was demonstrated using a Meta-Model. As shown in Table 13, the results achieved so far employing baseline characteristics and protein biomarkers are commendable on other measures. As a result of this, we present the results for a reduced collection of baseline characteristics such as education and gender, CDR-SSB, ADASQ4, RAVLT-I, RAVLT-L, RAVLT-PF, mPACCdigit, and mPACCtrailsB, along with only one protein biomarker (at a time). When only one specific protein biomarker (Aβ/Tau/Ptau) was included in addition to the aforesaid baseline characteristics, the results of the modeling are summarized in Table 14, whilst the findings of the Meta-Modeling are summarized in Table 15.

**Table 14:** Base model: baseline features + individual protein biomarker.

	**Baseline** + **A**β **protein biomarker**														
	**Without imputation**					**Imputation with mean**					**Model imputation**				
**Base model**	**Train Acc. (%)**	**Test Acc. (%)**	**Pr. (%)**	**Rec. (%)**	**F1-sc**.	**Train Acc. (%)**	**Test Acc. (%)**	**Pr. (%)**	**Rec. (%)**	**F1-sc**.	**Train Acc. (%)**	**Test Acc. (%)**	**Pr. (%)**	**Rec. (%)**	**F1-sc**.
LR	96.20	95.20	95.00	95.00	0.95	95.80	87.50	88.21	87.50	0.87	96.10	85.71	86.53	85.71	0.85
KNN	89.00	88.10	89.00	88.00	0.88	89.40	79.50	81.00	79.00	0.79	90.50	77.70	79.00	78.00	0.77
LDA	93.80	94.00	94.00	94.00	0.94	94.40	84.80	86.00	85.00	0.84	94.40	84.80	86.00	85.00	0.84
QDA	89.00	84.50	87.00	85.00	0.84	75.00	70.00	70.00	68.00	0.67	74.60	66.10	75.00	66.00	0.64
DT	96.40	94.05	94.42	94.05	0.94	100.00	86.00	86.00	86.00	0.86	98.20	86.00	86.00	86.00	0.85
RF	100.00	95.20	96.00	95.00	0.95	99.30	86.60	88.00	87.00	0.86	99.30	85.70	86.00	86.00	0.85
AB	100.00	95.20	96.00	95.00	0.95	99.60	88.30	90.00	89.00	0.89	99.60	88.40	89.00	88.00	0.88
PCA-LR	90.40	85.70	87.00	86.00	0.85	92.30	80.40	81.00	80.00	0.79	92.60	80.40	81.00	80.00	79.00
SVM-RBF	97.10	91.70	92.00	92.00	0.92	96.10	88.40	89.00	88.00	0.88	94.40	86.60	87.00	87.00	0.86
PC	90.00	85.70	87.00	86.00	0.85	90.80	80.40	81.00	80.00	0.80	88.70	77.70	79.00	78.00	0.77
ML-PC	48.80	45.70	24.00	49.00	0.32	60.00	57.70	79.00	59.00	0.51	62.70	57.10	47.00	57.00	0.47
EN	91.70	90.00	92.00	92.00	0.92	92.00	84.80	86.00	85.00	0.84	92.00	84.80	86.00	85.00	0.84
**Baseline** + **Tau protein biomarker**															
**Base model**	**Train Acc. (%)**	**Test Acc. (%)**	**Pr. (%)**	**Rec. (%)**	**F1-sc**.	**Train Acc. (%)**	**Test Acc. (%)**	**Pr. (%)**	**Rec. (%)**	**F1-sc**.	**Train Acc. (%)**	**Test Acc. (%)**	**Pr. (%)**	**Rec. (%)**	**F1-sc**.
LR	96.40	95.20	97.00	96.00	0.96	96.10	87.50	88.00	88.00	0.87	96.00	87.00	88.00	88.00	0.87
KNN	90.00	85.70	87.00	86.00	0.85	95.10	87.50	86.00	86.00	0.86	95.10	86.00	86.00	86.00	0.86
LDA	95.20	93.00	95.00	95.00	0.95	94.00	84.00	85.00	84.00	0.83	94.00	84.00	85.00	84.00	0.83
QDA	70.20	64.40	85.00	70.00	0.69	72.20	64.30	69.00	64.00	0.64	87.00	78.00	81.00	78.00	0.77
DT	96.20	95.40	94.42	94.05	0.94	96.10	85.00	86.00	85.00	0.84	96.10	85.00	86.00	85.00	0.84
RF	100.00	94.00	94.00	94.00	0.94	99.60	86.60	88.00	87.00	0.86	100.00	87.00	87.00	87.00	0.86
AB	100.00	92.00	92.00	92.00	0.92	96.50	84.00	84.00	84.00	0.83	99.60	83.00	83.00	83.00	0.82
PCA-LR	87.50	84.50	85.00	85.00	0.4	92.30	78.60	82.00	79.00	0.77	93.00	83.00	84.00	83.00	0.83
SVM-RBF	97.10	93.00	93.00	93.00	0.93	96.00	88.40	89.00	88.00	0.88	95.40	86.00	86.00	86.00	0.85
PC	91.80	89.30	90.00	89.00	0.89	90.50	86.00	86.00	86.00	0.86	89.10	79.00	79.00	79.00	0.78
ML-PC	69.20	66.00	51.00	67.00	0.58	70.10	61.60	44.00	62.00	0.51	70.40	64.30	47.00	64.00	0.54
EN	91.70	90.40	92.00	92.00	0.92	93.00	85.00	86.00	85.00	0.84	93.00	85.00	86.00	85.00	0.84
**Baseline** + **PTau protein biomarker**															
**Base model**	**Train Acc. (%)**	**Test Acc. (%)**	**Pr. (%)**	**Rec. (%)**	**F1-sc**.	**Train Acc. (%)**	**Test Acc. (%)**	**Pr. (%)**	**Rec. (%)**	**F1-sc**.	**Train Acc. (%)**	**Test Acc. (%)**	**Pr. (%)**	**Rec. (%)**	**F1-sc**.
LR	96.40	95.20	97.00	96.00	0.96	96.10	88.40	89.00	88.00	0.88	96.00	86.61	87.26	86.61	0.86
KNN	90.00	84.50	86.00	85.00	0.84	90.10	81.20	82.00	81.00	0.81	90.50	81.20	82.00	81.00	0.81
LDA	94.00	92.80	94.00	94.00	0.94	94.00	84.00	85.00	84.00	0.83	94.00	84.00	85.00	84.00	0.83
QDA	93.00	91.80	93.00	93.00	0.93	64.30	59.00	75.00	64.00	0.60	75.00	73.20	75.00	73.00	0.73
DT	96.20	94.05	94.42	94.05	0.94	96.10	88.00	86.00	85.00	0.84	96.10	85.00	86.00	85.00	0.84
RF	98.10	95.20	96.00	95.00	0.95	97.20	86.00	86.00	86.00	0.85	97.50	85.00	86.00	85.00	0.84
AB	100.00	95.20	96.00	95.00	0.95	100.00	86.00	86.00	86.00	0.85	100.00	86.00	86.00	86.00	0.86
PCA-LR	88.00	84.50	85.00	85.00	0.84	93.30	83.00	83.00	83.00	0.83	92.30	83.00	83.00	83.00	0.83
SVM-RBF	97.10	93.00	93.00	93.00	0.93	95.10	87.50	88.00	88.00	0.87	96.50	91.10	91.00	91.00	0.91
PC	88.10	87.00	90.00	88.00	0.88	90.50	86.00	86.00	86.00	0.86	91.20	84.00	85.00	84.00	0.84
ML-PC	72.10	70.00	52.00	68.00	0.59	54.50	51.10	32.00	54.00	40.00	69.00	60.00	42.00	60.00	0.50
EN	92.00	90.40	92.00	92.00	0.92	93.30	85.00	86.00	85.00	0.84	93.30	86.00	86.00	85.00	0.84

**Table 15:** Meta-model: baseline features + individual protein biomarker.

	**Without imputation**		**Imputation with mean**		**Model imputation**	
	**Train Acc. (%)**	**Test Acc. (%)**	**Train Acc. (%)**	**Test Acc. (%)**	**Train Acc. (%)**	**Test Acc. (%)**
**Baseline** + **A**β **protein biomarker**						
Accuracy	97.12	96.20	96.83	89.30	97.54	89.30
Precision (%)	97.00		90.00		89.00	
Recall (%)	96.00		89.00		89.00	
F-measure	0.96		0.89		0.89	
Hamming loss	0.06		0.13		0.14	
Jaccard index	0.89		0.78		0.75	
**Baseline** + **Tau protein biomarker**						
Accuracy	96.15	96.40	96.50	89.30	96.50	87.50
Precision (%)	97.00		87.50		90.00	
Recall (%)	97.00		86.61		89.29	
F-measure	0.96		0.86		0.89	
Hamming loss	0.05		0.13		0.12	
Jaccard index	0.87		0.77		0.81	
**Baseline** + **PTau protein biomarker**						
Accuracy	96.63	96.30	95.42	89.50	96.50	88.40
Precision (%)	97.00		88.00		89.00	
Recall (%)	97.00		87.50		88.00	
F-measure	0.96		0.87		0.88	
Hamming loss	0.05		0.12		0.13	
Jaccard index	0.89		0.88		0.88	

Comparing Tables 12, 14 results for Base Models demonstrated that when only the Tau protein biomarker was evaluated, the no-imputation strategy offered more accurate findings with a 95.40% accuracy using the Decision Trees classifier. When only the Aβ/Tau/PTau protein biomarker and baseline parameters were included, the imputation with mean approach achieved an accuracy of 88.00% (Table 14). When compared to Table 12, which contained performance results for the specified baseline features and the three protein indicators, it was determined that the Logistic Regression (Multinomial) classifier had an accuracy of 86.00% when employing a similar imputation strategy. When only the Aβ protein biomarker was included in addition to baseline features, the accuracy of the model imputation technique on the AdaBoost classifier was 88.40%, respectively. When compared to Table 14, the Logistic Regression (Multinomial) classifier had an accuracy of 87.30% for the Linear Regression imputation strategy.

Table 15 presents the Meta-Model built for all baseline features along with a single protein biomarker. It demonstrated that the three meta-models' performance accuracy ranged from 87.00% to 96.40% in three of the cases (Table 15). Additionally, the precision, recall, F1-score, and classification errors were all noteworthy. This suggested that the developed model performed efficiently on both employed techniques i.e., when all protein biomarkers were included and when a single protein biomarker was used to diagnose the response variable as CN, AD, or MCI, which was a significant indicator. When compared to the results in Table 13, this implied that the Meta-Model resulted in a modest increase in outcomes when Aβ, Tau, and PTau were taken into account in the mean imputation technique. However, the Meta-Model (Table 13) achieved an accuracy of 97.60% for the no-imputation technique, which was greater than the no-imputation method in Table 15 for all Aβ/Tau/PTau protein biomarkers.

### 3.4 For baseline + medication + protein (C = 4)

The selected features after applying the feature selection technique for controller 4 included: Education, Gender, CDR-SB, ADAS13, ADASQ4, MMSE, RAVLT-I, RAVLT-L, RAVLT-PF, DIGITSCOR, LDETOTAL, mPACCdigit, Ventricles, Entorhinal, Aβ, Tau, PTau, Ethnicity, Race category, Married status, APOE4, Blood thinner, Calcium, Cholesterol, Cognitive, and Vitamin D. The results of the modeling for the base ML models are summarized in Table 16. Table 17 presents the results of the Meta-Model construction process as well as the metrics used to evaluate performance.

**Table 16:** Performance result on base model for C = 4.

	**Without imputation**					**Imputation with mean**					**Model imputation**				
**Base model**	**Train Acc. (%)**	**Test Acc. (%)**	**Pr. (%)**	**Rec. (%)**	**F1-sc**.	**Train Acc. (%)**	**Test Acc. (%)**	**Pr. (%)**	**Rec. (%)**	**F1-sc**.	**Train Acc. (%)**	**Test Acc. (%)**	**Pr. (%)**	**Rec. (%)**	**F1-sc**.
LR	98.10	95.20	95.00	95.00	0.95	98.00	87.50	88.00	88.00	0.88	97.50	88.40	89.00	88.00	0.88
KNN	87.00	82.10	83.00	82.00	0.82	88.00	80.00	82.00	79.00	0.79	87.30	78.60	80.00	79.00	0.78
LDA	95.20	94.00	94.00	94.00	0.94	97.00	83.00	85.00	83.00	0.83	97.00	82.10	83.00	82.00	0.82
QDA	58.70	53.60	68.00	54.00	0.44	55.00	54.00	54.00	54.00	0.45	55.00	54.50	58.00	54.00	0.46
DT	96.40	94.05	94.42	94.05	0.94	96.10	85.00	86.00	85.00	0.84	98.00	85.00	86.00	85.00	0.84
RF	100.00	95.20	96.00	95.00	0.95	100.00	85.00	85.00	85.00	0.84	100.00	87.50	88.00	87.50	0.87
AB	96.40	94.05	94.42	94.05	0.94	100.00	87.50	88.00	88.00	0.88	100.00	87.00	87.00	87.00	0.86
PCA-LR	91.70	91.00	92.00	92.00	0.92	96.10	84.80	85.00	85.00	0.84	92.30	82.10	83.00	82.00	0.82
SVM-RBF	100.00	88.10	88.00	88.00	0.88	96.10	86.60	87.00	87.00	0.86	99.30	88.40	88.00	88.00	0.88
PC	91.80	89.30	90.00	89.00	0.89	92.30	82.10	83.00	82.00	0.82	92.30	78.00	81.00	78.00	0.76
ML-PC	70.20	70.00	55.00	68.00	0.60	60.00	55.40	50.00	45.00	46.00	73.00	60.00	43.00	59.00	0.49
EN	94.70	90.50	92.00	90.00	0.91	93.70	85.70	86.00	86.00	0.85	94.40	84.80	85.00	85.00	0.84

**Table 17:** Result: meta-model (C = 4).

	**Without imputation**		**Imputation with mean**		**Model imputation**	
	**Train Acc. (%)**	**Test Acc. (%)**	**Train Acc. (%)**	**Test Acc. (%)**	**Train Acc. (%)**	**Test Acc. (%)**
Accuracy	98.00	96.20	97.53	88.40	98.24	89.30
Precision (%)	97.00		88.21		90.00	
Recall (%)	96.00		88.00		89.00	
F-measure	0.96		0.87		0.89	
Hamming loss	0.05		0.12		0.12	
Jaccard index	0.90		0.78		0.79	

As demonstrated in Table 16, when employed without imputation on the test set, the Logistic Regression (Multinomial) algorithm produced an accuracy of 95.20%. In comparison to the Meta-Model, it attained a high accuracy of 96.20% when the identical approach was used. When the mean imputation approach was applied, the base model's accuracy was 87.50%. Following that, an imputation with a mean technique with an accuracy of 88.40% was used to demonstrate the enhanced accuracy for a Meta-Model. Additionally, model imputation yielded an accuracy of 88.40% on the Logistic Regression (Multinomial) classifier for a Base Model, while the Meta-Model yielded an accuracy of 89.30% for the same applied approach. As shown in Table 17, the findings thus far based on baseline characteristics, medication, and protein biomarkers were notable in terms of additional performance evaluation criteria.

### 3.5 Comparative analysis

**Figure 6:**
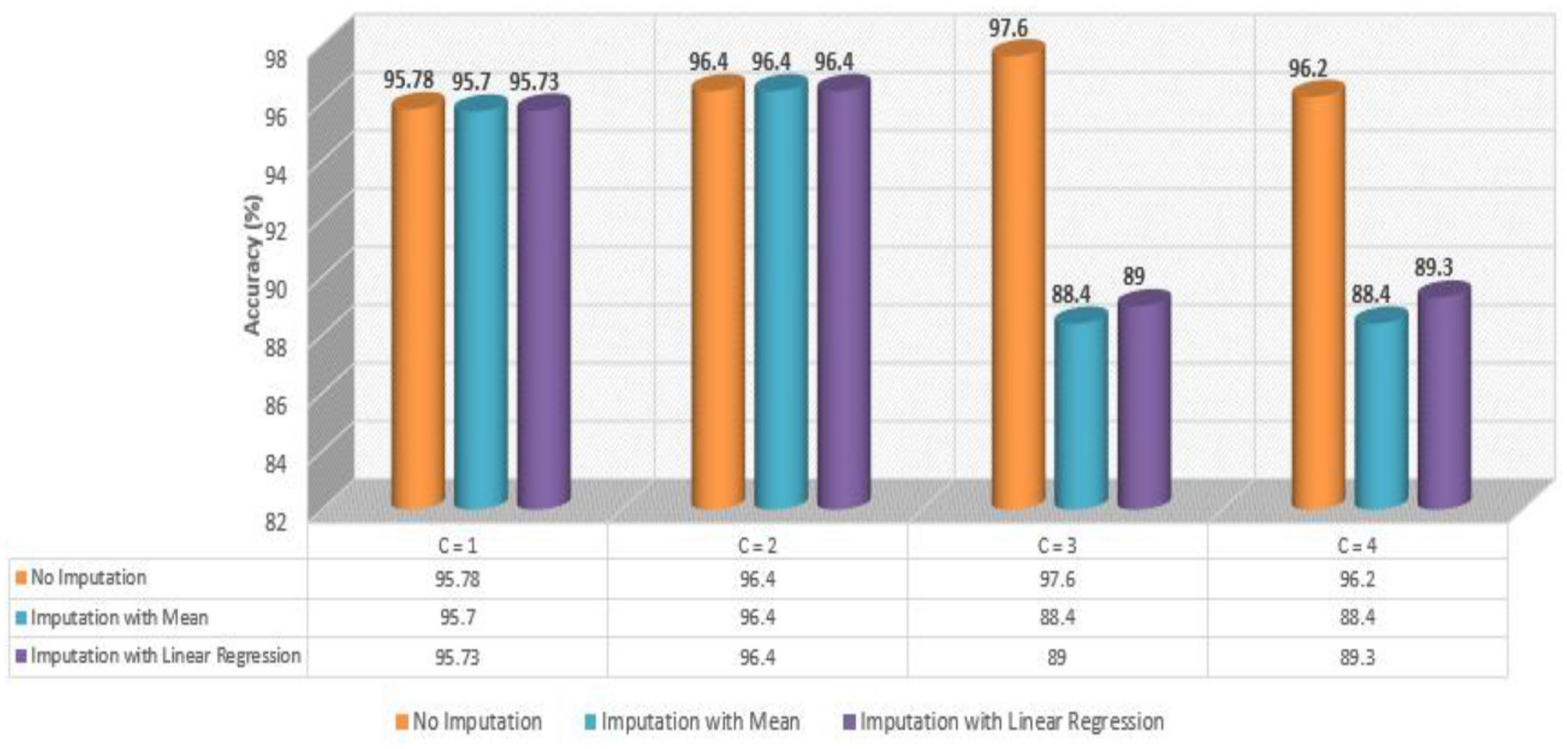
Comparative analysis of meta-models.

Figure 6 presents a comparative analysis of all four developed Meta-Models based on performance accuracy. We can comprehend the following from Figure 6: the highest accuracy of 97.60% was shown by no imputation approach when the controller C = 3 (baseline + protein); for controller C = 4 (baseline + drug + protein), the no imputation approach gave an improved accuracy of 96.20% amongst the three employed techniques; for C = 2 (baseline + drug), the accuracy of 96.40% for all the three applied approaches was observed; and when C = 1 (baseline data), the accuracy for all the three methods was found to be close to 96.0% respectively.

## 4 Discussion

The timely and precise diagnosis of AD is critical to reducing the consequences of this disorder, whose incidence has been on the rise. The application of ML can enhance the clinical diagnosis of AD in its early stages and provide valuable insights into research on this damaging and progressive disease. Early Alzheimer's disease was diagnosed in this study by considering characteristics associated with specific AD medications such as calcium, vitamin D supplements, blood thinner medicines, cholesterol-lowering drugs, and cognitive drugs, including a substantial protein biomarker (Aβ, tau, and ptau) as a predictor. To simulate the MRI-based data, we proposed a hybrid-clinical model. The diagnostic group in this study comprised five categories. We designed a pipeline that integrated exhaustive approaches for detecting AD across a broad range of input values and parameters. We evaluated the association between the diagnosis of AD and the use of several drugs in particular. Aiming to better understand how Alzheimer's is diagnosed, we looked at the importance of three cerebrospinal fluid biomarkers: tau, ptau, and Aβ. The proposed design generated four Meta-Models for four different sets of criteria. The diagnostic criteria were established based on baseline features, baseline and drug characteristics, baseline and protein features, and baseline, drug, and protein features. The developed model incorporated a range of methodologies, including data collection and integration, multi-step data pre-processing, feature selection, development of machine learning models employing wide-ranging methods, optimization and analysis of results, measuring of test performance, construction of Meta-Model, generalized performance metrics, and comparative analysis.

Our results indicate that patients' age, education, gender, CDR_SB, ADAS13, ADASQ4, MMSE, RAVLT-I, RAVLT-L, RAVLT-PF, TRABSCOR, LDETOTAL, mPACCdigit, Ventricles, WholeBrain, Hippocampus, Entorhinal, Fusiform, MidTemp, ICV, ethnicity, race category, marital status, and APOE4 are associated with a higher likelihood of being diagnosed with AD based on our analysis of the baseline visit data. In addition, our results reveal that CSF biomarkers, tau, ptau, and Aβ, when added to the baseline model, could be the significant predictors. We were able to attain a maximum accuracy of 97.60% for baseline and protein data without any imputation technique. We observed that the constructed model functioned effectively when all five drugs were included, as well as when any single drug was used for the diagnosis of the response variable (CN, AD, or MCI). Interestingly, the constructed Meta-Model worked well when all three protein biomarkers were included, as well as when a single protein biomarker was utilized to diagnose the response variable. Thus, our developed model not only has the potential to aid clinicians and medical professionals in advancing Alzheimer's diagnosis but also serves as a valuable starting point for future research into AD and other neurodegenerative disorders. With further refinement and exploration, it could pave the way for innovative diagnostic techniques and therapeutic interventions in the field of neurology.

Researchers are increasingly employing machine learning and deep learning techniques in their work to classify and evaluate patients and the associated risks and predict treatment outcomes. One area where these methods have been particularly useful is in the classification of neurodegenerative conditions caused by AD, as well as their different stages, using imaging-based detection. Additionally, researchers have constructed automated pipelines that employ feature extraction approaches based on a range of biomarker methodologies to improve the quality of their findings (Shukla et al., 2023). ML models have become ubiquitous in real-time clinical applications, diagnostics, and the treatment of AD. Numerous recent studies (as reported in Table 18) have integrated MRI data into ML models for predicting AD as evidenced by the works of Gopi et al. (2020), Khan and Zubair (2020), Liu et al. (2020), Diogo et al. (2022), Kavitha et al. (2022), Khan and Zubair (2022b), and Uddin et al. (2023).

**Table 18:** Comparison with the state-of-the-art approaches.

**Author(s)**	**Proposed method**	**Dataset**	**Performance**
**Proposed approach**	**Hybrid-clinical model (majority voting and majority stacking)**	**ADNI**	**97.60% accuracy**
Uddin et al. (2023)	Voting method (Models comprising: GaussianNB, Decision Tree, Random Forest, XGBoost, Voting Classifier, and GradientBoost)	OASIS	96% accuracy
Kavitha et al. (2022)	Decision Tree, Random Forest, Support Vector Machine, Gradient Boosting, and Voting	OASIS	83%
Diogo et al. (2022)	Linear Support Vector Machine, Decision Tree, Random Forest, Extremely Randomized Tree, Linear Discriminant Analysis, Logistic Regression, Logistic Regression Classifier with Stochastic Gradient Descent Learning	ADNI and OASIS	90.6% balanced accuracy
Khan and Zubair (2022a,b)	Hybrid Model (Models comprising: Logistic Regression, GaussianNB, Support Vector Machine, Decision Trees, Random Forest, Extreme Gradient Boosting)	ADNI	95.12% accuracy
Gaetani et al. (2021)	LASSO-based logistic model	Laboratory of Clinical Neurochemistry, Department of Medicine and Surgery, University of Perugia (Perugia, Italy)	AUC score: 0.906
Khan and Zubair (2020)	Random Forest, Extra Trees, Decision Trees, NuSVC, Logistic RegressionCV, AdaBoost, Gradient Boosting, GaussianNB, RidgeClassifierCV, KNN	OASIS	87% accuracy
Liu et al. (2020)	Logistic RegressionCV, Linear SVC, Decision Tree, Bagging, MLP	Dem@Care FP7 project (Speech data)	86.1% accuracy
Gopi et al. (2020)	Pruned decision trees (J48)	OASIS	88.7% accuracy
Stamate et al. (2019)	Deep Learning (DL), Extreme Gradient Boosting (XGBoost) and Random Forest (RF)	European Medical Information Framework for Alzheimer's Disease Biomarker Discovery Cohort	AUC score: 0.87

Ensuring optimal accuracy for cognitive assessments in the context of AD continues to be a pressing challenge despite ongoing efforts. To bridge this gap, a novel clinical-hybrid model has been introduced to enhance the accuracy of Alzheimer's detection. To demonstrate the improvements and potential contributions of our new model in making cognitive tests more accurate for individuals with AD, Table 18 presents a comparison between our proposed method and prior research. However, it is essential to highlight that the datasets, the number of patients in each research, the classifiers used, and the modeling technique are all highly distinct, making direct comparison challenging.

Recent years have witnessed significant advances in research, most notably the discovery of biomarkers (especially brain imaging technologies) that enable the diagnosis and monitoring of AD-related processes months, years, and even decades before clinical problems appear. Alzheimer's disease biomarkers are divided into two types: early biomarkers, which measure amyloid accumulation in the brain (e.g., PET imaging, CSF amyloid), and late biomarkers, which measure neurodegeneration [e.g., structural MRI, fluorodeoxyglucose-positron emission tomography (FDG PET), CSF tau]. Few recent studies have found that AD biomarkers are associated with cognitive decline including Stamate et al. (2019) and Gaetani et al. (2021), as presented in Table 18.

Biomarkers are widely exploited in the diagnostic framework and help in designing appropriate therapy (when available), albeit these are largely intended for research use. There are two types of biomarkers: those that directly influence the pathology of AD, like the A beta-amyloid (Aβ) and tau proteins; and those that provide an indirect or non-specific indication of the disorder by locating indices of neuronal damage, which are considered to be the main cause of AD. While these indicators are generally related to Alzheimer's disease, they have been related to other types of illnesses as well. The concomitant presence of both proteins in an individual indicates a strong case of AD.

In the present circumstances, both advanced-stage AD and dementia are considered incurable. It could be attributed to the failure in the early stage of the disease. Currently, the goal of treatment is to reduce the course of the disease and also to control its symptoms. While this is extremely difficult, it is achievable to some extent if the disease is detected reasonably early. Treatments focus on symptom management, such as cognitive and psychological difficulties, as well as behavioral difficulties; environmental alteration to enable patients to do everyday activities more effectively; and caregiver support, such as family and friends.

The CSF analysis also provides information regarding blood-brain barrier damage and inflammatory diseases that resemble or make a contribution to dementia (Blennow et al., 2010). Nonetheless, more recently, approaches used in determining levels of biomarkers such as Aβ1-42, pTau, and total tau (tTau) have been established in investigating essential Alzheimer's disease pathology. The levels of Aβ1-42 are decreased in AD, although tTau (a more general sign of neuronal degeneration) and pTau (a more specific diagnostic for AD) have been found to get elevated during the progression of AD. Current research suggests that a low Aβ1-42 value combined with a high pTau or tTau value provides the most diagnostic specificity (Hansson et al., 2006). In the absence of established pathology, it is difficult to define reference ranges as well as cut points for these parameters. A lack of uniformity in CSF gathering and processing has been reported to make it more difficult (Mattsson et al., 2009; Bartlett et al., 2012). However, efforts are being made to resolve these concerns, and CSF analysis has been inducted into Alzheimer's disease research diagnostic guidelines (Dubois et al., 2014).

Moreover, the imminent application of plasma biomarkers, including plasma Aβ and tau, holds promise for enhancing AD diagnosis (Sun et al., 2022). With advancements in research and the potential approval of disease-modifying therapies targeting MCI-AD or AD-dementia, the imperative for earlier and more accurate diagnosis of AD becomes increasingly critical (Cummings, 2019; Arafah et al., 2023). The advent of disease-modifying drugs highlights the importance of identifying individuals at risk of developing AD in preclinical stages, paving the way for proactive interventions aimed at delaying or preventing disease progression (Crous-Bou et al., 2017).

## 5 Conclusion

This study presents a notable improvement in early AD detection through the integration of machine learning, statistical modeling, and biomarker indicators. The optimized predictive models demonstrate a robust diagnostic framework that takes into account various factors, including patient drugs, protein biomarkers, and baseline features. The proposed hybrid-clinical model and the in-depth analysis of the correlations between demographic, clinical, and biomarker variables and AD diagnosis underscore its potential to revolutionize clinical detection. The high level of accuracy obtained with both baseline and protein data serves as validation for the efficacy of the developed models. The comprehensive pipeline and Meta-Models designed for various diagnostic criteria, offer a versatile approach for clinicians. Additionally, the comparative analysis with existing studies highlights the novel contributions of this research while acknowledging the challenges in direct comparisons due to variations in datasets and methodologies. Overall, this study not only provides valuable insights into Alzheimer's diagnosis but also sets a basis for future research into neurodegenerative disorders, emphasizing the essential role of advanced technologies in transforming diagnostic approaches. It could be hypothesized that incorporating patients' drugs or protein biomarkers into an ML model to supplement clinical diagnoses could enhance diagnostic accuracy. Our improved model with drug features could mitigate the challenge of diagnosing the early stages of AD when other symptoms are not as easily observable, which remains a pervasive issue for both clinical and research professionals.

## Data Availability

The original contributions presented in the study are included in the article/supplementary material, further inquiries can be directed to the corresponding author.

## Footnotes

^1^*ADNI | About*. Available at: http://adni.loni.usc.edu/about/.

^2^*Alzheimer's Dementia Causes, Risk Factors | Research Center*. Alzheimer's Association. Available at: https://www.alz.org/alzheimers-dementia/what-is-alzheimers/causes-and-risk-factors.
